# Are Appearances Deceiving? Morpho-Genetic Complexity of the *Eumerus tricolor* Group (Diptera: Syrphidae) in Europe, with a Focus on the Iberian Peninsula†

**DOI:** 10.3390/insects14060541

**Published:** 2023-06-10

**Authors:** Pablo Aguado-Aranda, Antonio Ricarte, Zorica Nedeljković, Scott Kelso, André P. W. van Eck, Jeffrey H. Skevington, María Ángeles Marcos-García

**Affiliations:** 1 Research Institute CIBIO (Centro Iberoamericano de la Biodiversidad), Science Park, University of Alicante, Ctra. San Vicente del Raspeig s/n, 03690 San Vicente del Raspeig, Alicante, Spain; pablo.aguado@ua.es (P.A.-A.); zoricaned14@gmail.com (Z.N.); marcos@ua.es (M.Á.M.-G.); 2 Canadian National Collection of Insects, Arachnids and Nematodes, Agriculture and Agri-Food Canada, K.W. Neatby Building, 960 Carling Avenue, Ottawa, ON K1A 0C6, Canada; scott.kelso@agr.gc.ca (S.K.); jhskevington@gmail.com (J.H.S.); 3 BioMongol Foundation, Korte Hoefstraat 30, 5046 DB Tilburg, The Netherlands; andrevaneck@freedom.nl

**Keywords:** hoverfly, Merodontini, phenotypic diversity, male genitalia, COI, systematics

## Abstract

**Simple Summary:**

Hoverflies are a diverse group (6200+ species) of two-winged flies that provide important ecosystem services (e.g., plant pollination and regulation of populations of pest insects). The hoverfly genus *Eumerus* has caught the attention of scientists and entomology amateurs due to its high number of species worldwide (250+) and its wide range of body sizes and shapes. Nevertheless, some species show a similar overall appearance making both their separation from each other and the study of their classification/biology difficult. Thus, the aim of the present work is to update the knowledge of the *Eumerus* hoverflies in the Iberian Peninsula by assessing the diversity of the *Eumerus tricolor* group under an integrative approach (i.e., combining different techniques and data sources). We found and described two new species from Spain and provided the most comprehensive identification key for all known European species of this *Eumerus* group.

**Abstract:**

*Eumerus* Meigen, 1822 is one of the largest Syrphidae genera in the Palaearctic Region, with the highest levels of taxonomic diversity found in the *Eumerus tricolor* species group. Despite its high diversity, the interspecific levels of morphological variability can be low. Additionally, some species may show certain levels of intraspecific variability. Hence, species delimitation may become challenging. In this work, we assessed the diversity of the *E. tricolor* group in the Iberian Peninsula through an integrative analysis of nomenclature, morphology and the 5′ (COI-5′) and 3′ (COI-3′) end regions of the Cytochrome *c* oxidase subunit I gene. Two new species, *Eumerus ancylostylus* Aguado-Aranda & Ricarte sp. n. and *Eumerus petrarum* Aguado-Aranda, Nedeljković & Ricarte sp. n., were described, and their intra- and interspecific variations discussed. In addition, the first barcodes of Iberian members of the *E. tricolor* group were obtained, and the distribution ranges of all species were mapped within the study area. The systematic position of the new species is discussed based on the resulting COI-based trees. The male genitalia of *Eumerus hispanicus* van der Goot, 1966 and *Eumerus bayardi* Séguy, 1961 were studied and illustrated. A lectotype was designated for *Eumerus lateralis* (Zetterstedt, 1819). An updated dichotomous key for all known European species of the *E. tricolor* group is provided. The egg of *E. petrarum* sp. n. is also described.

## 1. Introduction

The Syrphidae is one of the best-known Diptera families, especially in Europe [1]. Recent works report a great morphological and genetic complexity in this family at different taxonomic levels (e.g., [2,3]). Thus, species delimitation becomes difficult in taxonomically complex groups [4], especially when cryptic species (i.e., species with identical or very similar morphology) are involved [5]. Cases of cryptic speciation are found in various genera of hoverflies (e.g., [6,7]). Furthermore, phylogenetically-related cryptic species (i.e., sibling species *sensu* Knowlton [8]) have also been reported within the Syrphidae, mainly for genera with high species richness, such as *Merodon* Meigen, 1803 [9].

The genus *Eumerus* Meigen, 1822 (Eristalinae: Merodontini) is one of the most diverse among hoverflies worldwide [10]. In total, 11 species groups are known to be present in the European continent, of which the *Eumerus tricolor* (Fabricius, 1798) group is the most species-rich [11]. This group was proposed by Chroni et al. [12] based on COI mitochondrial DNA. Grković et al. [13] defined it morphologically in order to include species with the following combination of features: large and, predominantly, black body, but abdomen usually with red parts; basoflagellomere square-shaped or oval, more or less striated and with a flattened ellipsoidal area distally; in males, eyes dichoptic or slightly holoptic; anterior surstylar lobe of male genitalia poorly developed; interior accessory lobe of male genitalia wing-shaped and densely pilose. This diagnosis has been updated in later studies as some characters (e.g., the coloration of body hairs) appeared to be variable amongst species [14]. Within the *E. tricolor* group, the *Eumerus binominatus* Hervé-Bazin, 1923 subgroup was defined by Grković et al. [15]. All species of this subgroup share the apomorphic characters of the *E. tricolor* group but differ in the shape and length of the legs, which are conspicuously thin and slender. The number of species belonging to the *E. tricolor* group is continually increasing in the Palaearctic Region (e.g., [11,16]). Regarding their conservation, only the European species of the *E. tricolor* group have been assessed according to IUCN Red List Categories and Criteria [17].

The Ibero-Balearic region (mainland Portugal and Spain, plus Andorra, Gibraltar and the Balearic Islands) hosts a great diversity of hoverflies, with 420+ species recorded [18,19,20,21]. The genus *Eumerus* is particularly diverse in the Ibero-Balearic region, with 37 species reported, of which 7 are endemic [22,23,24]. Nowadays, 9 species groups and a number of “individual species” (not assigned to a species group) have representation in the Ibero-Balearic area, with the *E. tricolor* group having the highest number of species included [22,24]: *Eumerus azabense* Ricarte & Marcos-García in Ricarte et al., 2018, *Eumerus grallator* Smit in Grković et al., 2019a, *Eumerus grandis* Meigen, 1822, *Eumerus hispanicus* van der Goot, 1966, *Eumerus larvatus* Aracil, Grković & Pérez-Bañón in Aracil et al., 2023, *Eumerus ovatus* Loew, 1848, *Eumerus sabulonum* (Fallén, 1817), *Eumerus tarsalis* Loew, 1848 and *E. tricolor*.

Recent findings of new species of *Eumerus* in the Iberian Peninsula [14,23,24,25] suggest that the *Eumerus* diversity from this area might be underestimated, especially for the highly diverse *E. tricolor* group, which has been poorly explored in this region. Thus, in the framework of an ongoing taxonomic revision of the Ibero-Balearic *Eumerus*, the objectives of the present work are to assess the diversity of this species group and to explore the taxonomy and systematic position of its species through an integrative approach.

## 2. Materials and Methods

### 2.1. Study Area

The Ibero-Balearic area, with an extension of more than 600,000 km^2^, is located in south-western Europe. Two biogeographical regions are represented in the Iberian territory: the Euro-Siberian and the Mediterranean [26]. The first one is restricted to the northernmost areas encompassing rainy, mountainous landscapes where the Pyrenees and the Cantabrian Mountain Range are found. Otherwise, the Mediterranean region includes a vast mosaic of habitats [27]. In the framework of the present study, two Pyrenean enclaves of Spain (‘valle de Camprodrón’, in Girona province and ‘Refugio de Belagua,’ in Navarra province) were surveyed, as well as various localities in south-eastern Spain, mainly along the Baetic System: ‘Sierra de Aitana,’ ‘Sierra del Maigmó,’ and ‘Sierra de Mariola’ in Alicante province, ‘Sierra de Gádor,’ in Almería province and ‘Sierra Nevada,’ in Granada province.

### 2.2. Examined Material

A total of 219 adult specimens of 11 species of the *E. tricolor* group were studied in the present work. New material was collected in different Iberian localities (see Study area) and European countries by hand net, Malaise trap and pan trap. We also studied eggs of *Eumerus* aff. *sabulonum* laid within a plastic tube where a female was introduced upon capture in the field. Material deposited in the following entomological collections was also examined: the private collection of André P.W. van Eck (AET, Tilburg, The Netherlands); ‘Colección Entomológica de la Universidad de Alicante’ (CEUA-CIBIO, Alicante, Spain); the Canadian National Collection of Insects, Arachnids and Nematodes (CNC, Ottawa, ON, Canada); Melbourne Museum (MMV, Museums Victoria, Australia); ‘Museo Nacional de Ciencias Naturales’ (MNCN, Madrid, Spain); Museum of Zoology (MZLU, University of Lund, Sweden); Naturalis Biodiversity Centre (NBC, Leiden, The Netherlands); private collection of Sander Bot (PSB, Haren, The Netherlands); the private collection of Jeroen van Steenis (PJSA, Amersfoort, The Netherlands).

All specimens were databased in an excel table. A unique bar code label was assigned to the new material added to the CEUA-CIBIO collection. In the examined material lists, data from different labels of the same specimen are separated by a single forward slash (/). Collection acronyms are indicated in brackets. A semicolon (;) is used for separating the collection code labels of different specimens with the same locality data. Additional information not specified in the labels is indicated in curly brackets ({}). The information on the examined specimens is detailed in a supplementary file (see Supplementary material S1), except for that of the new species, which is included in the main text document. A distribution map with all known Iberian records of species of the *E. tricolor* group was produced with the software QGIS v3.22.16 [28], for which both published and new data were considered. Taking into consideration the diagnosis of the *E. tricolor* group [13], a comprehensive review of the species descriptions available in the literature was performed to produce a preliminary list of all putative Palaearctic species of this group.

### 2.3. Morphological Study

All species were, at the first stage, identified with the available dichotomous keys for *Eumerus* [15,29,30,31]. For the undescribed species not suiting the species included in the keys, their taxonomic statuses were investigated. In the new species descriptions, first, a diagnosis was provided and second, a detailed morphological description of males (based on the holotype) and females, if available. Then, species distribution, biology and special remarks of the new species are indicated. To complete the new species descriptions, the following measures were taken on several adult specimens (‘*n*’) with the software Leica Application Suite X (LAS X) ^®^ v3.0.4.16529: Body length (‘*l*’); basoflagellomere ratio (length:width; see figure 4a of Aguado-Aranda et al. [23]); and the maximum width of the vertical triangle (‘*vtw*’) measured at a mid-point on the ocellar triangle. Male genitalia were dissected and prepared following Ricarte et al. [32] and then stored in glycerine in plastic microvials. All photographs of the specimens and male genitalia were taken with a Leica DFC 450 camera attached to a Leica M205 C binocular microscope. Male genitalia of *Eumerus bayardi* Séguy, 1961, *E. hispanicus*, *E. sabulonum* and *Eumerus* aff. *sabulonum* were hand drawn from printed photographs.

For eggs of *Eumerus* aff. *sabulonum*, a detailed description of their external surface was undertaken from images of scanning electron microscopy (SEM). The eggs were fixed and preserved in ethanol 70%. They were dehydrated using an ethanol/water series of 2 baths of 70% for 5 min each, followed by one bath of 80%, 90% and 96% ethanol for 5 min each and 2 baths in absolute ethanol of 5 min each. The dehydrated eggs were mounted on aluminum stubs with a double-sided adhesive carbon tape and sputter-coated in a Quorum 150 T ES Plus with a 30 nm layer of platinum. The photographed egg was not uniformly turgid. The images were taken with a Jeol JSM-ITH500HT at 3.0 kV accelerating voltage.

Morphological terminology used in the adult descriptions follows Thompson [33] except for the term “hair/s,” which is used in replacement of “pilis/pile,” as well as the terms “fossette” and “notopleural sulcus” which follow Doczkal & Pape [34]. The terminology for male genitalia follows Doczkal [35], and for egg follows Chandler [36]. For those illustrations cited from the literature (not shown in the present paper), ‘figure’ (lower case) is used, while ‘Figure’ (upper case) is used for those which are original from this work (shown in the present paper).

### 2.4. Molecular Study

DNA was extracted both from 1 mesoleg and 1 metaleg of 2 males of *Eumerus* aff. *grandis*, 3 specimens (2 males and 1 female) of *E. grallator*, 2 specimens (1 male and 1 female) of *E. hispanicus*, 8 specimens (5 males and 3 females) of *Eumerus* aff. *sabulonum*, 5 specimens (4 males and 1 female) of *E. sabulonum*, 1 male of *E. tarsalis* and 2 specimens (1 male and 1 female) of *E. tricolor*. DNA of one male of *Eumerus* aff. *grandis* (code CEUA00017798) and two males of *E. grandis* were extracted from a wing. The NZY Tissue gDNA Isolation kit, following the manufacture’s protocol for animal tissues, was used for two males of *Eumerus* aff. *grandis* and all specimens of *E. grallator*, *E. hispanicus*, *E. sabulonum*, *Eumerus* aff. *sabulonum*, *E. tarsalis* and *E. tricolor*. Otherwise, the DNA of 1 male of *Eumerus* aff. *grandis* (code CEUA00017798), 2 males of *E. bayardi* and 2 males of *E. grandis* was extracted using the QIAGEN DNeasy Blood and Tissue kit with some modifications in the manufacturer’s protocol.

PCR amplifications of the 5′ (COI-5′) and 3′ (COI-3′) end regions of the Cytochrome *c* oxidase subunit I gene were performed using universal and custom primers (Table 1). The COI-5′ fragments “A,” “B” and “C” were amplified for “older” specimens (collection date previous to the year 2000) in which DNA is heavily fragmented and “recent” specimens of which the complete COI-5′ was not achieved to amplify. All amplifications were carried out in a total volume of 25 µL containing 1 × of Buffer reaction, 0.4 mM of dNTPs, 0.2 µM of each primer, 0.65–2 mM of MgCl_2_ and 1–2 units of DNA polymerase. Thermocycler conditions followed those used by Grković et al. [37] and Grković et al. [25] for COI-5′, and Chroni et al. [12], but with annealing at 48 °C, for COI-3′. Otherwise, the PCR profile for the barcodes of *E. bayardi*, all fragments of COI-5′ and COI-3′ (specimen vouchers CEUA_S71–72, S83–84, S86–87, S93 and S231–232) consisted of an initial denaturalization of 3 min at 94 °C; 45 cycles of 45 s at 94 °C, annealing of 45 s at 45 °C and 1 min at 72 °C; with a final elongation step of 5 min at 72 °C.

All PCR products were visualized with an electrophoresis process in a 1–1.5% agarose gel, except those of *E. bayardi,* which were visualized and purified in an E-Gel CloneWell 0.8% agarose gel (Life Technologies Corporation, Austin, TX, USA). Purifications of COI-5′ fragments and COI-3′ (vouchers above indicated) products followed the USB^®^ ExoSAP-IT^®^ method (Affymetrix, Inc., Santa Clara, CA, USA). Sequencing reactions of the fragments, COI-5′ of *E. bayardi* and COI-3′ products (vouchers above indicated) were carried out with the BigDye^TM^ Terminator v3.1 Cycle Sequencing kit (Thermo Fisher Scientific, Inc., Waltham, MA, USA). The rest of the PCR products were purified and sequenced at Macrogen (Macrogen, Inc., Seoul, Republic of Korea).

The sequences were edited by eye with the program Sequencher v5.4.6 (Gene Codes Corporation 2017, Ann Arbor, MI). Then, COI-5′ and COI-3′ sequences of *Eumerus alpinus* Rondani, 1857, *Eumerus minotaurus* Claussen & Lucas, 1988, *Eumerus sogdianus* Stackelberg, 1952 and at least one male and female specimen of each European species of the *E. tricolor* group available at the public repositories GenBank and BOLD [38] were downloaded (see Supplementary Material S2). Alignments were performed manually and checked with the program AliView v1.25 [39]. The final COI-5′ and concatenated (COI-5′+COI-3′) matrices had lengths of 569 and 1289 bp, respectively. Then, Neighbor-Joining (NJ) and Maximum Likelihood (ML) analyses for both datasets were carried out in MEGA7 [40] with 1000 bootstrap replications using the Maximum Likelihood Composite and General Time Reversible (GTR) models, respectively. A GTR+G model (with gamma distribution) was used for the COI-5′ matrix, and a GTR+G+I model (with invariant sites) was used for the COI (COI-5′+COI-3′) matrix. The resulting trees were rooted based on a *Xanthogramma citrofasciatum* (De Geer, 1776) sequence.

**Table 1:** List of primers used in the molecular study of the *E. tricolor* species group.

Gene Region	Primer	Sequence	Reference
COI-5′ (complete)	LCO1490	5′-GGTCAACAAATCATAAAGATATTGG-3′	[41]
	HCO2198	5′-TAAACTTCAGGGTGACCAAAAAATCA-3′	
COI-5′ (“A”)	Heb-F	5′-GGTCAACAAATCATAAAGATATTGG-3′	[41]
	1762-R	5′-CGDGGRAADGCYATRTCDGG-3′	Kelso (*in prep.*)
COI-5′ (“B”)	342-F	5′-TGTAAAACGACGGCCAGTGGDKCHCCNGAYATRGC-3′	Kelso (*in prep.*)
	1976-R	5′-GWAATRAARTTWACDGCHCC-3′	
COI-5′ (“C”)	1957-F	5′-GGDATWTCHTCHATYYTAGG-3′	Kelso (*in prep*.)
	780-R	5′-CCAAAAAATCARAATARRTGYTG-3′	[42]
COI-3′	780-F	5′-CARCAYYTATTYTGATTTTTTGG-3′	Kelso (*in prep.*)
	C1-J-2183 (*Jerry*)	5′-CAACATTTATTTTGATTTTTTGG-3′	[43]
	TL2-N-3014 (*Pat*)	5′-TCCAATGCACTAATCTGCCATATTA-3′	

## 3. Results

### 3.1. Integrative Approach

**Figure 1:**
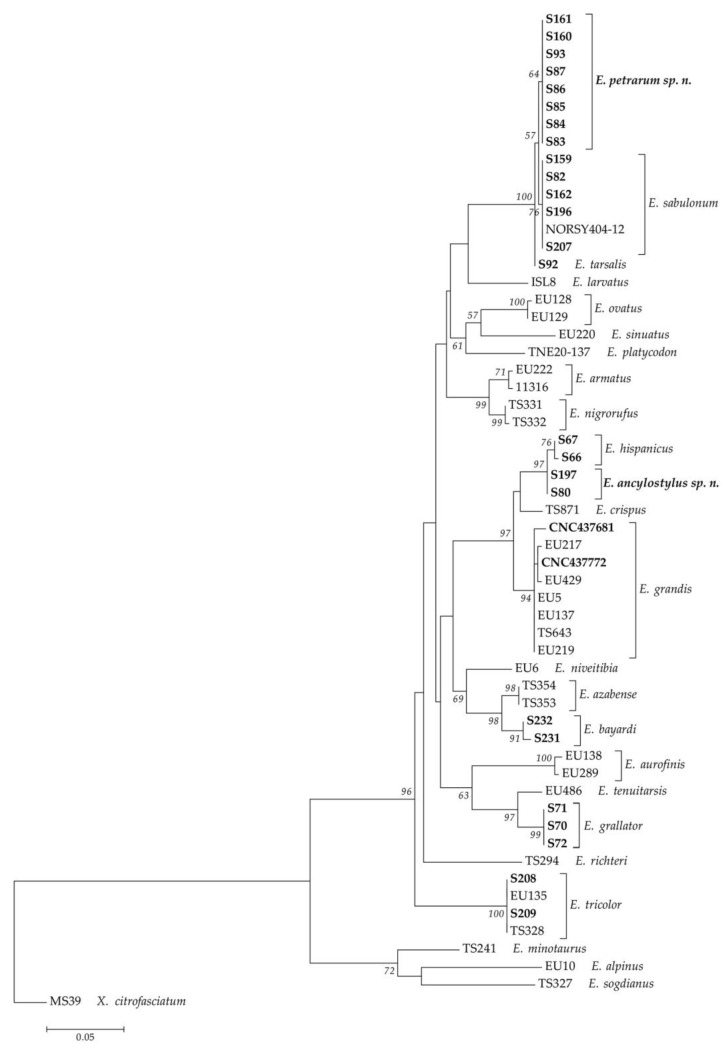
Maximum Likelihood tree based on COI-5′. DNA vouchers of specimens analyzed for this work are highlighted in bold. Bootstrap values > 50 are shown near nodes. Branch lengths are measured in the number of substitutions per site.

**Figure 2:**
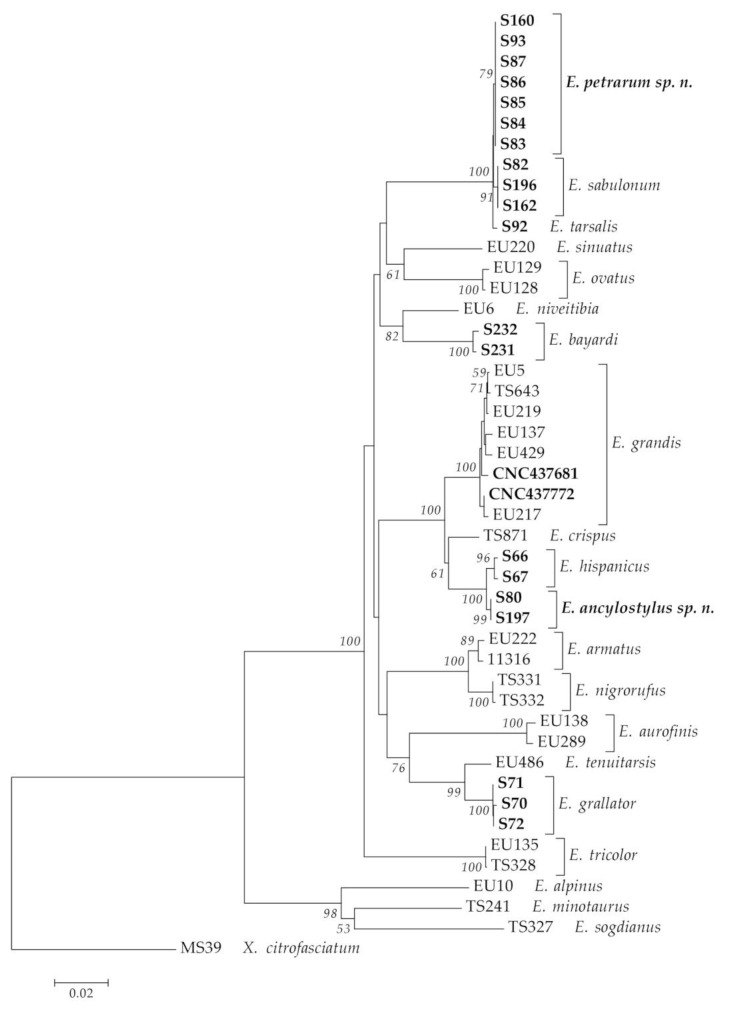
Neighbor-Joining tree based on COI (COI-5′+COI-3′). DNA vouchers of specimens analyzed for this work are highlighted in bold. Bootstrap values > 50 are shown near nodes. Branch lengths are measured in the number of substitutions per site.

As a result of the literature review, 66 species of the *E. tricolor* group were found to occur in the Palaearctic Region (Table 2). Regarding the Ibero-Balearic area, 128 new specimens were collected of the following species: 2 males of *Eumerus* aff. *grandis*, 1 male of *E. azabense*, 1 male of *E. bayardi*, 26 specimens of *E. grallator* (25 males and 1 female), 4 specimens of *E. hispanicus* (3 males and 1 female), 31 specimens of *E. sabulonum* (27 males and 4 females), 41 specimens of *Eumerus* aff. *sabulonum* (36 males and 5 females), 5 males of *E. tarsalis* and 17 specimens of *E. tricolor* (13 males and 4 females). In the molecular analyses, 21 species of the *E. tricolor* group were considered in the COI-5′ dataset and 17 species in the COI (COI-5′+COI-3′) dataset (see Supplementary Material S2). Apart from those of the undescribed species (*Eumerus* aff. *sabulonum*, *Eumerus* aff. *grandis*), we generated the first known COI sequences for the following species: *E. bayardi*, *E. grallator*, *E. hispanicus*, *E. sabulonum* and *E. tarsalis*. In addition, we also obtained new COI sequences for *E. grandis* and COI-5′ for *E. tricolor*. All resulting trees had virtually the same topologies (Figure 1 and Figure 2). The COI-based trees showed the highest bootstrap values (>70) for the two new species that clustered in single clades (Figure 2), supporting our morphological hypotheses. Therefore, based on the combination of the nomenclatural, morphological and molecular evidence, the new species were characterized.

**Table 2:** Preliminary list of the Palaearctic species of the *E. tricolor* group according to the reviewed literature, with an indication of their type locality (only country/region). For the European species, the category assigned in the European Red List of Hoverflies is also indicated. *Eumerus afrarius* Séguy, 1961 and *Eumerus lateralis* (Zetterstedt, 1819) are omitted from this list due to their uncertain taxonomic status. The species of the *E. binominatus* subgroup are indicated with an asterisk. Legend: Not Evaluated (NE), Least Concern (LC), Vulnerable (VU), Endangered (EN), Critically Endangered (CR).

Species	Type Locality	IUCN Red List Category
*E. alajensis* Peck, 1966	Kyrgyzstan	⸺
*E. ancylostylus* Aguado-Aranda & Ricarte, sp. n.	Spain	⸺
*E. arctus* van Steenis in Grković et al., 2021	Switzerland	NE
*E. arkadii* Mutin in Mutin & Barkalov, 1999	Russia	⸺
*E. arkitensis* Peck, 1969	Kyrgyzstan	⸺
*E. armatus* Ricarte & Rotheray in Ricarte et al., 2012	Greece	VU
*E. armenorum* Stackelberg, 1960	Armenia	⸺
*E. atricolorus* Gilasian & van Steenis in Gilasian et al., 2020	Iran	⸺
*E. aurofinis* Grković, Vujić & Radenković in Grković et al., 2016	Greece	EN
*E. azabense* Ricarte & Marcos-García in Ricarte et al., 2018	Spain	CR
*E. badkhyziensis* Mutin, 2019	Turkmenistan	⸺
*E. bayardi* Séguy, 1961	France	⸺
*E. binominatus* Hervé-Bazin, 1923*	Kazakhstan	⸺
*E. brevipilosus* Gilasian & van Steenis in Gilasian et al., 2020	Iran	⸺
*E. chekabicus* Gilasian & van Steenis in Gilasian et al., 2020	Iran	⸺
*E. coeruleithorax* Peck, 1969	Kazakhstan	⸺
*E. coeruleus* (Becker, 1913)	Iran	⸺
*E. compertus* Villeneuve in Villeneuve & Gauthier, 1924	Tunisia	⸺
*E. crispus* Vujić & Grković in Grković et al., 2021	Serbia	NE
*E. falsus* Becker, 1922	Syria	⸺
*E. grallator* Smit in Grković et al., 2019a*	Spain	VU
*E. grandis* Meigen, 1822	Europe	LC
*E. grisescens* Becker, 1921	Russia	⸺
*E. hispanicus* van der Goot, 1966	Spain	VU
*E. hissaricus* Stackelberg, 1949	Tajikistan	⸺
*E. jacobsoni* Becker in Becker & Stein, 1913	Iran	⸺
*E. kazanovzkyae* Paramonov, 1927	Azerbaijan	⸺
*E. kirgisorum* Peck, 1966	Kyrgyzstan	⸺
*E. kopetdagicus* Barkalov & Mutin, 2022	Turkmenistan	⸺
*E. larvatus* Aracil, Grković & Pérez-Bañón in Aracil et al., 2023	Spain	⸺
*E. leleji* Mutin, 2016	Khakassia	⸺
*E. longitarsis* Peck, 1979*	Tajikistan	⸺
*E. lunatus* (Fabricius, 1794)	North Africa	⸺
*E. mucidus* Bezzi, 1921	Africa	⸺
*E. nigrifacies* Becker, 1921	Russia	⸺
*E. nigrorufus* Grković & Vujić in Grković et al., 2021	Montenegro	NE
*E. niveitibia* Becker, 1921	Bulgaria	VU
*E. ovatus* Loew, 1848	Europe	EN
*E. ovoformus* Gilasian & van Steenis in Gilasian et al., 2020	Iran	⸺
*E. palaestinensis* Stackelberg, 1949	Israel	⸺
*E. pamirorum* Stackelberg, 1949	Tajikistan	⸺
*E. pavlovskii* Stackelberg, 1964	Armenia	⸺
*E. persarum* Stackelberg, 1961	Iran	⸺
*E. persicus* Stackelberg, 1949	Iran	⸺
*E. petrarum* Aguado-Aranda, Nedeljković & Ricarte, sp. n.	Spain	⸺
*E. pilosipedes* Gilasian & van Steenis in Gilasian et al., 2020	Iran	⸺
*E. platycodon* Choi & Hong in Choi et al., 2021	South Korea	⸺
*E. richteri* Stackelberg, 1960	Azerbaijan	⸺
*E. rubrum* Grković & Vujić in Grković et al., 2017	Greece	EN
*E. rubescens* Villeneuve, 1912	Syria	⸺
*E. rufipilus* Peck, 1969	Kyrgyzstan	⸺
*E. rufomaculatus* Peck, 1966	Kyrgyzstan	⸺
*E. ryzhik* Barkalov & Mutin, 2022	Uzbekistan	⸺
*E. sabulonum* (Fallén, 1817)	Sweden	LC
*E. selevini* Stackelberg, 1949	Kazakhstan	⸺
*E. sinuatus* Loew, 1855	Austria	EN
*E. tadzhikorum* Stackelberg, 1949*	Tajikistan	⸺
*E. tarsalis* Loew, 1848	Europe	EN
*E. tauricus* Stackelberg, 1952	Ukraine	EN
*E. tenuitarsis* Grković & Vujić in Grković et al., 2019a*	Greece	CR
*E. tricolor* (Fabricius, 1798)	Switzerland	LC
*E. turcmenorum* Paramonov, 1927	Turkmenistan	⸺
*E. urartorum* Stackelberg, 1960	Armenia	⸺
*E. ursiculus* Stackelberg, 1949	Tadzhikistan	⸺
*E. ussuriensis* Stackelberg, 1952	Russia	⸺
*E. vallicolus* Gilasian & van Steenis in Gilasian et al., 2020	Iran	⸺

### 3.2. New Species Descriptions

**Figure 3:**
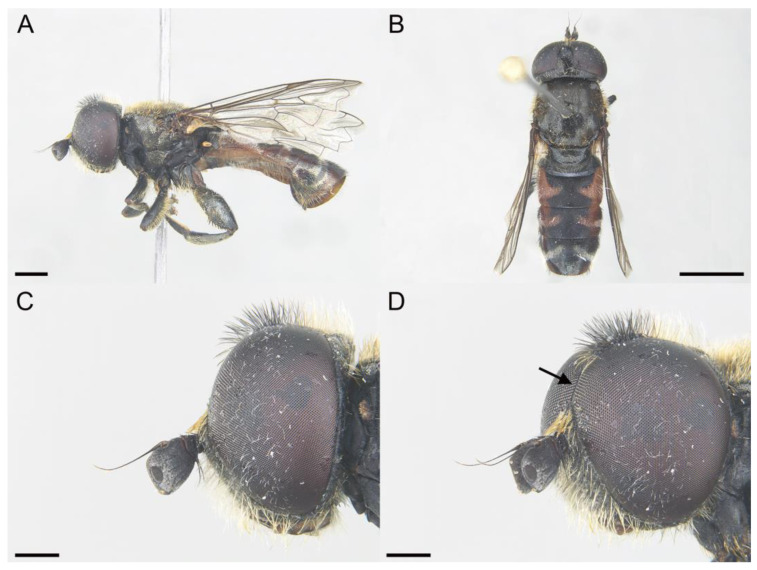
*Eumerus ancylostylus* sp. n., male (holotype): (**A**) Habitus, lateral view. (**B**) Habitus, dorsal view. (**C**) Head, lateral view. (**D**) Eye contiguity (indicated by an arrow). Scale bars = (**A**) 1 mm; (**B**) 2 mm; (**C**,**D**) 500 µm.

**Figure 4:**
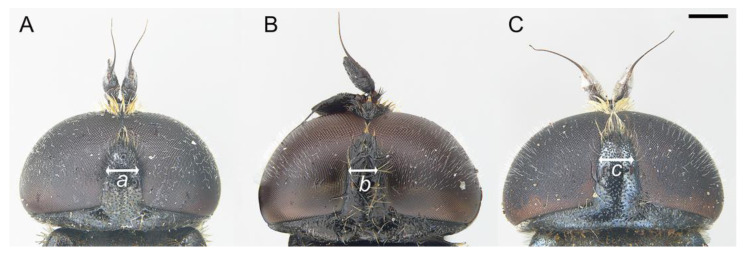
Male, vertical triangle: (**A**) *E. ancylostylus* sp. n. (holotype). (**B**) *E. grandis* (specimen from Montenegro). (**C**) *E. lateralis* (lectotype). Vertical triangle width (mm): *a* = 0.58, *b* = 0.43, *c* = 0.5. Scale bar = 500 µm.

**Figure 5:**
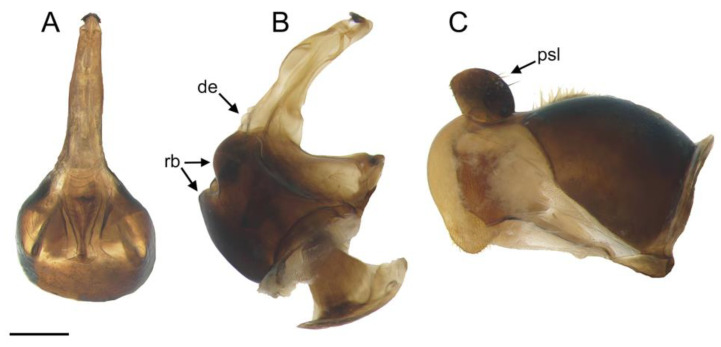
*Eumerus ancylostylus* sp. n. (holotype), male genitalia: (**A**) Hypandrium, ventral view. (**B**) Hypandrium, lateral view (right side). (**C**) Epandrium, lateral view (right side). Legend: de, dentate expansion; psl, posterior surstylar lobe; rb, rounded bulges. Scale bar = 250 µm.

**Figure 6:**
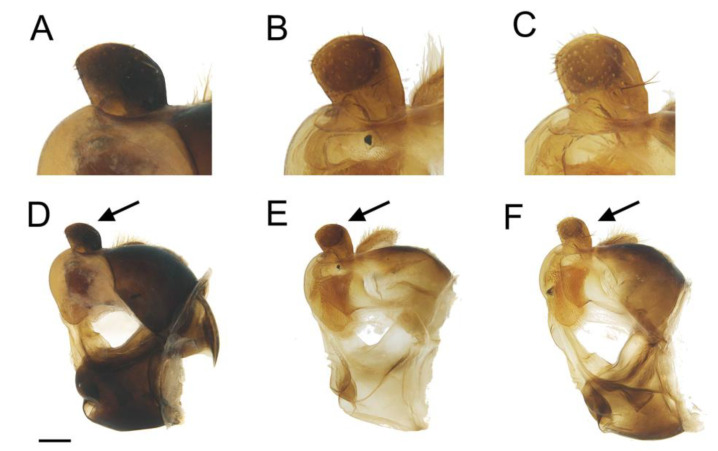
Surstylus, lateral view (right side): (**A**) *E. ancylostylus* sp. n. (holotype). (**B**) *E. grandis* (specimen from Montenegro). (**C**) *E. lateralis* (lectotype). Male genitalia, lateral view (right side): (**D**) *E. ancylostylus* sp. n. (**E**) *E. grandis*. (**F**) *E. lateralis*. An arrow indicates the posterior surstylar lobe, which is enlarged in the images above. Scale bar (**D**–**F**) = 250 µm.

*Eumerus ancylostylus* Aguado-Aranda & Ricarte, sp. n. (Figure 3, Figure 4A, Figure 5 and Figure 6A,D)

= *Eumerus* aff. *grandis*

urn:lsid:zoobank.org:act:19E89FBD-90F2-4187-A933-F19D846B8CEE

Holotype

SPAIN • ♂—ESPAÑA, Girona, Setcases, camino del depósito de agua, 1317 m, 30-VII-2020, Leg.: A. Ricarte/*Eumerus grandis* Meigen, 1822, Det. Z. Nedeljković & A. Ricarte/DNA CEUA_S80/CEUA00108275 (CEUA-CIBIO) {as *E. grandis*, in [44]}.

Paratypes

SPAIN • 1♂—ESPAÑA, Girona, Setcases, camino del depósito de agua, 1317 m, 02-VIII-2020, Leg.: Z. Nedeljković/*Eumerus grandis* Meigen, 1822, Det. Z. Nedeljković & A. Ricarte/DNA CEUA_S197/CEUA00108274 (CEUA-CIBIO) {as *E. grandis*, in [44]} • 1♂—Valle de Casares—LE, 4-VI-87, Mª. A. Marcos-García {leg}/*Eumerus annulatus* (Panzer), Det.: Mª. A. Marcos-García/*Eumerus grandis* Meigen, 1822, Det.: A. Ricarte, 2009/DNA CEUA_S275/CEUA00017798 (CEUA-CIBIO) {as *E. annulatus*, in [45]}.

Diagnosis. This species can be distinguished by the following combination of features (only males, unknown female): large body size (>9.5 mm); eyes hairy and touching along a line (Figure 3C,D); wide vertical triangle (0.58–0.61 mm) (Figure 4A); basoflagellomere trapezoidal in shape; mesonotum with moderately long, golden yellow hairs (Figure 3A), which are of two lengths intermixed on the posterior part, with the shortest hairs surpassing half the length of the longest ones; posterior surstylar lobe arched anteriorly (Figure 6A). This species is most similar to *E. grandis* (see Taxonomic notes).

Etymology. The specific epithet ‘ancylostylus’ originates from the combination of the Latinised Greek words ‘ankylos’ (curved) and ‘stylos’ (stylus, of the male genitalia) and refers to the curved/round anterior part of the posterior surstylar lobe in male.

Description. MALE (*holotype*). Measurements (mm): *l* = 9.56; *vtw* = 0.58. *Head.* Eyes contiguous along a line. Eye hairs moderately short and disperse (Figure 3C,D). Face and frontal triangle black; face grey pollinose. Frontal triangle and face covered with dense, golden hairs. Ocellar triangle isosceles. Vertical triangle black; anterior half (including ocellar triangle) with long, erect and black hairs. Apex and posterior half of the vertical triangle covered with whitish-yellow hairs (Figure 3C,D). Occiput black, with whitish yellow hairs which become shorter (than those on the vertical triangle) and whiter toward the laterals; occiput slightly grey pollinose along eye margin and covered in short, black hairs. Scape and pedicel black; pedicel with black hairs ventrally, longer than those on the dorsal side of the scape. Basoflagellomere trapezoidal in shape (length:width ratio = 1:1), slightly striated radially and black; basoflagellomere with a round fossette at the distal margin (Figure 3C). Pedicel and basoflagellomere sparsely white pollinose (pollinosity more obvious under artificial white lighting). Arista black and bare. *Thorax.* Mesonotum and pleura black. Scutum covered with golden yellow hairs, slightly shorter than those on the vertical triangle; hairs on the posterior half of the scutum almost uniformly long, with the shortest hairs surpassing half of the length of the longest ones; scutum with two medial, white pollinose vittae slightly exceeding the transverse suture; width of the vittae less than 1/8 of the total width of scutum. Notopleural sulcus absent. Disc of scutellum with golden yellow hairs of the same length as that on the scutum; posterior margin of the scutellum with small teeth-like protuberances, each bearing a long black hair apically. Posterior anepisternum, anterior anepimeron and katepisternum on its postero-lateral and ventral areas bearing densely-arranged long golden yellow hairs. Katatergum with a discrete bunch of white hairs. Pleuron grey pollinose except the anterior anepimeron posterolaterally, the upper half of the posterior anepimeron and the katepimeron anterolaterally. Metasternum covered with long, yellowish-white hairs. Femora black; apices of pro- and mesofemora light brown. Tibiae black; pro- and mesotibiae dark brown basally. Tarsi black dorsally and yellow ventrally. Profemur with long, directed backward, golden yellow hairs on its posterior side. Mesofemur with long, directed backward, golden yellow hairs on its posterior side, which are curved at their apex. Pro- and mesofemur with few long, directed backward, black hairs intermixed with the yellow ones on their posterior sides. Metacoxa densely covered with long and white hairs anteriorly and bare posteriorly. Metafemur with dense and yellow hairs, with black hairs postero-apically; hairs on ventral side of the metafemur at least twice the length of the subapical spinae. Metafemur with an anteroventral row of nine spinae and a posteroventral row of six spinae apically. Metatibia with a basoventral ridge covered with short, reclined setae. Wing membrane extensively microtrichose; posterior margin of the wing with dense, short and brown hairs; margin of ventral calypter with rather long and yellow hairs; hairs on the margin of dorsal calypter shorter than those on the ventral calypter; halter brownish yellow. *Abdomen.* Tergum I black. Terga II-IV black, lateral sides reddish-orange; terga II-IV with a pair of slightly curved, white pollinose maculae. Terga II-IV covered with short, semi-reclined, black hairs. Tergum II with white hairs on lateral margins (hairs at the anterior corners rather long). Tergum III covered with short and black hairs on lateral margins (hairs on the basal part of the maculae white). Tergum IV with short and black hairs on the anterior half of the lateral margins, with white hairs on the posterior half of lateral margins. Sterna I-IV dark brown; anterior margin of sternum I black. Sternum IV flat (without projections); posterior margin of sternum IV slightly V-shaped. Sterna II and III covered with white hairs. Sternum IV covered with short, reclined, white hairs. *Genitalia.* Epandrium with a simple posterior surstylar lobe, straight posteriorly and rounded anteriorly, and slightly inclined in the direction opposite to cerci. Cercus with long hairs. Hypandrium with two large, round bulges and three little, almost rounded, hyaline expansions at the base (Figure 5B).

FEMALE. Unknown.

**Figure 7:**
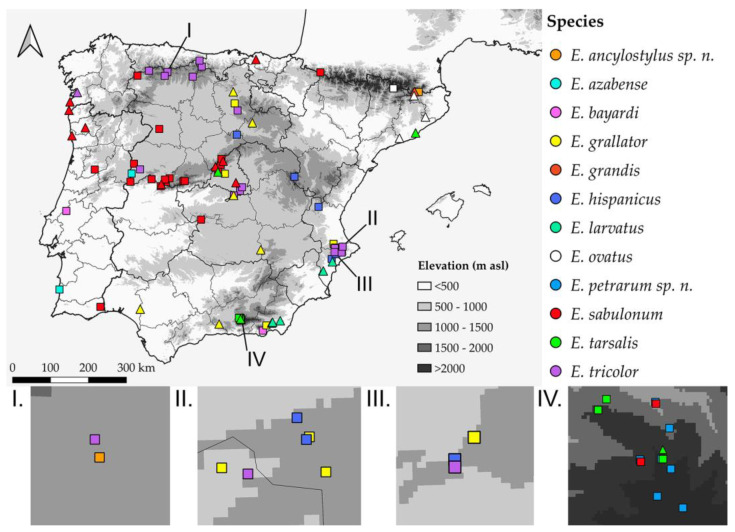
Distribution ranges of the Iberian members of the *E. tricolor* species group. Confirmed records are indicated with a square. Unconfirmed published records are indicated with a triangle.

Distribution. The Pyrenees and Cantabrian mountain ranges, in the Spanish provinces of Girona and León (Figure 7).

Biology. Adults fly from June to early August at altitudes of over 1300 m asl. The predominant vegetation in the Cantabrian locality of *E. ancylostylus* sp. n. was mowing meadows and hazelnut woods [46]. The specimens from the Pyrenees were found in a valley, on a side mountain track, amongst vegetation consisting of herbs, scrubs and mixed woodland, and relatively close to a river [44].

Taxonomic notes. The new species resembles morphologically *E. grandis,* which was described from an undetermined number of specimens from ‘Europe’ (unknown precise locality). The type material of *E. grandis* is lost [11]. According to Peck [47], *E. grandis* has two synonyms: *Eumerus annulatus*, originally described as *Syrphus annulatus* Panzer, 1798, and *Eumerus varius* Meigen, 1822. *Syrphus annulatus sensu* Panzer was revealed to be a primary homonym of *S. annulatus* Fabricius, 1798, recently synonymized with *Merodon natans* (Fabricius, 1794) [48]. Grković et al. [11] confirmed the status of *E. annulatus* (Panzer, 1798) as a synonym of *E. grandis*. In addition, *E. varius* was excluded from the list of synonyms of *E. grandis* since the examined male of *E. varius* from the supposed type series was actually *E. tricolor*. This male was designated as lectotype of *E. varius* in order to stabilize this species concept [11].

*Eumerus ancylostylus* sp. n keyed out as *E. grandis* in the keys for the south-eastern European species of the *E. tricolor* group [11] and in those of the *Eumerus* from Switzerland and surrounding parts of central Europe [30]. Moreover, the new species keyed out as *E. annulatus*, later described by Meigen as *E. grandis* [11], in the Stackelberg’s key for the Palaearctic species of *Eumerus* [31]. Nevertheless, the new species differs from *E. grandis* in the basoflagellomere ratio, which is wider in *E. ancylostylus* sp. n. (1:1–1.1, *n* = 3) than in *E. grandis* (1:0.8–0.9, *n* = 3), the width of the vertical triangle, which is wider in *E. ancylostylus* sp. n. (Figure 4A,B), the length of the hairs on posterior half of the scutum, which are all almost of equal length in *E. ancylostylus* sp. n. but clearly different in length in *E. grandis* (short hairs do not exceed half of the length of the longest hairs) and the shape of the posterior surstylar lobe, rounded anteriorly in *E. ancylostylus* sp. n. but more square-shaped in *E. grandis* (Figure 6A,B). In addition, *E. ancylostylus* sp. n. differs clearly from *E. grandis* in COI sequences.

**Figure 8:**
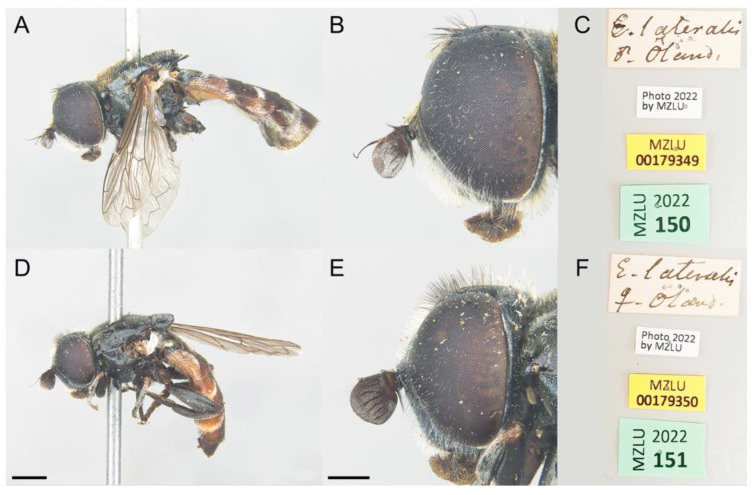
*Eumerus lateralis*, male (lectotype): (**A**) Habitus, lateral view. (**B**) Head, lateral view. (**C**) Original labels. Female (paralectotype): (**D**) Habitus, lateral view. (**E**) Head, lateral view. (**F**) Original labels. Scale bars = (**A**,**D**) 1 mm; (**B**,**E**) 500 µm.

We also examined the type material of *Eumerus lateralis* (Zetterstedt, 1819) (Figure 8), described under the genus *Pipiza* Fallén, 1810, because Meigen [49] stated in his description of *E. grandis*: “Whether this species is *Pipiza lateralis* Falléni (v. Kon. Vet. Ak. Handl. 1819. St. I. Nro. 38) I cannot state for lack of an accurate description of it”. After examination of the type material of *E. lateralis* (1 male and 1 female), we confirmed that all were conspecific and the male differed from *E. ancylostylus* sp. n. in the basoflagellomere ratio, which is wider in *E. ancylostylus* sp. n. (1:1–1.1, *n* = 3) than in *E. lateralis* (1:0.8), the width of the vertical triangle, slightly wider (0.1 mm) in the new species (Figure 4A,C), the length of the hairs on posterior half of the scutum, which are all almost of equal length in *E. ancylostylus* sp. n. but different in length in *E. lateralis* (short hairs are at most as long as half the length of the longest hairs) and the shape of the posterior surstylar lobe, which is square-shaped in *E. lateralis*, as in *E. grandis* (Figure 6B,C). The male from the type series of *E. lateralis* was designated as lectotype to stabilize the morphological concept of this species. Fresh material of *E. lateralis* from the type locality or close was not available to us. Given the similarity among these species and in the absence of molecular data on *E. lateralis*, we cannot confirm if *E. lateralis* is the same species as *E. grandis* or not. Nevertheless, based on the morphological similarities, *E. lateralis* and *E. grandis* may actually be the same species. Thus, fresh material of *E. lateralis* should be studied in the future to clarify the taxonomic status of this species.

**Figure 9:**
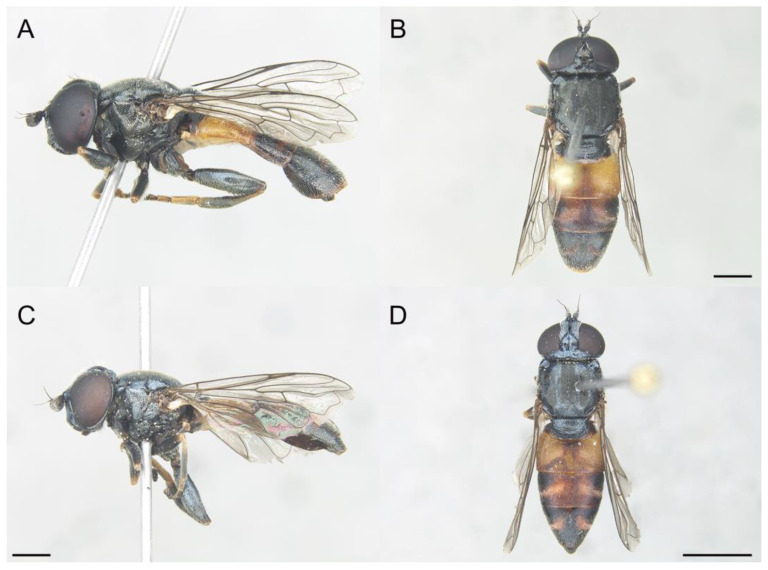
*Eumerus petrarum* sp. n., male (holotype), habitus: (**A**) Lateral view. (**B**) Dorsal view. Female (paratype), habitus: (**C**) Lateral view. (**D**) Dorsal view. Scale bars = (**A**–**C**) 1 mm; (**D**) 2 mm.

**Figure 10:**
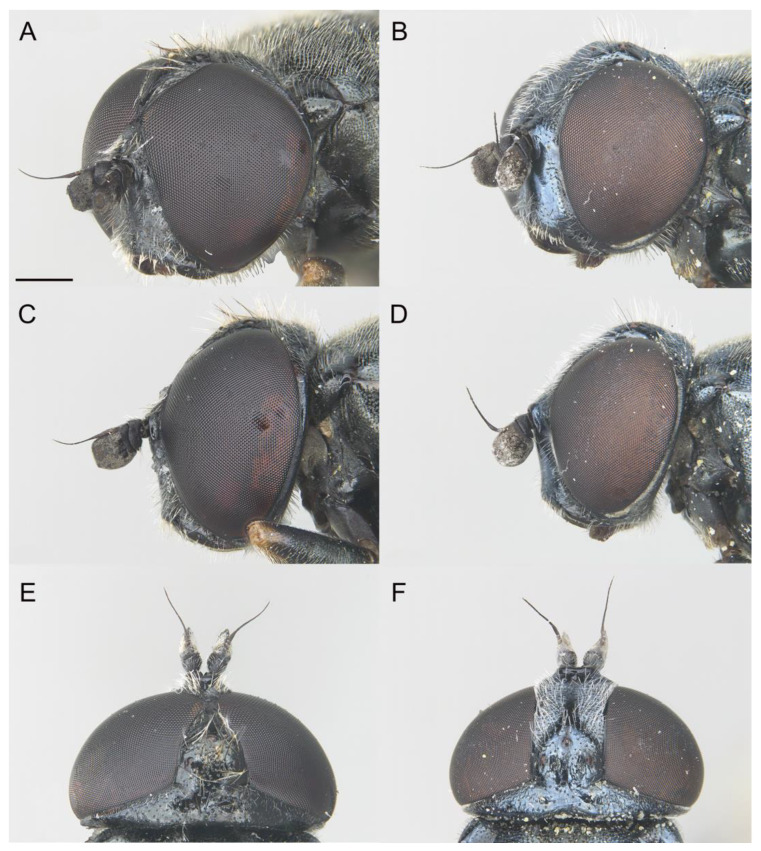
*Eumerus petrarum* sp. n., head: (**A**) Male (holotype). (**B**) Female (paratype). Head, lateral view: (**C**) Male. (**D**) Female. Head, dorsal view: (**E**) Male. (**F**) Female. Scale bar = 500 µm.

**Figure 11:**
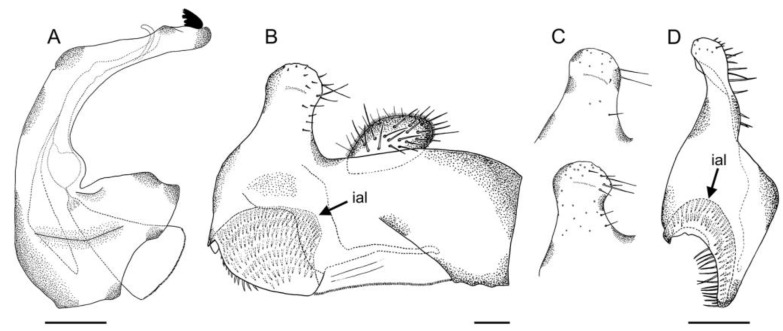
*Eumerus petrarum* sp. n., male genitalia (holotype): (**A**) Hypandrium, lateral view (right side). (**B**) Epandrium, lateral view (right side). (**C**) Surstylus, intraspecific variation. (**D**) Epandrium, ventral view. Legend: ial, interior accessory lobe. Scale bars = (**A**,**D**) 250 µm; (**B**,**C**) 100 µm.

**Figure 12:**
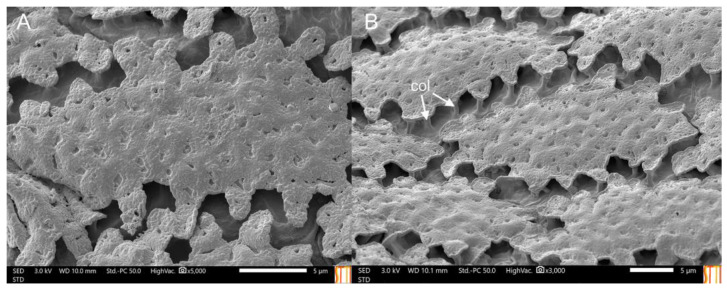
*Eumerus petrarum* sp. n., egg: (**A**) Detail of the chorionic sculpture. (**B**) Units conforming to the chorionic sculpture. Units may touch each other in some parts because the egg was not uniformly turgid. Legend: col, columns.

*Eumerus petrarum* Aguado-Aranda, Nedeljković & Ricarte, sp. n. (Figure 9, Figure 10, Figure 11 and Figure 12)

= *Eumerus* aff. *sabulonum*

urn:lsid:zoobank.org:act:DBC2C8BA-B818-49F7-BB91-856B966E51B6

Holotype

SPAIN • ♂—ESPAÑA (GR), Sierra Nevada, Monachil, camino a Laguna de las Yeguas, 25-VI-2021, 2890 m, Leg.: A. Ricarte/DNA CEUA_S85/CEUA00109899 (CEUA-CIBIO).

Paratypes

SPAIN • 3♂♂—ESPAÑA (GR), Sierra Nevada, Monachil, camino a Laguna de las Yeguas, 25-VI-2021, 2890 m, Leg.: A. Ricarte/CEUA00109901; 00109904; 00109904 (CEUA-CIBIO) • 5♂♂—ESPAÑA (GR), Sierra Nevada, Monachil, camino a Laguna de las Yeguas, 25-VI-2021, 2890 m, Leg.: P. Aguado Aranda/DNA CEUA_S160/CEUA00109906; 00109905 (CEUA-CIBIO); 00109902; 00109903 (AET); 00109900 (MNCN) • 4♂♂—ESPAÑA (GR), Sierra Nevada, Monachil, Sendero al Borreguil de San Juan, 22-VI-2021, 2540 m, Leg.: A. Ricarte/DNA CEUA_S83/CEUA00109885; 00109886; 00109891; 00109892 (CEUA-CIBIO) • 1♀—ESPAÑA (GR), Sierra Nevada, Monachil, Sendero al Borreguil de San Juan, 22-VI-2021, 2540 m, Leg.: A. Ricarte/DNA CEUA_S86/CEUA00109912 (CEUA-CIBIO) • 3♂♂—ESPAÑA (GR), Sierra Nevada, Monachil, Sendero al Borreguil de San Juan, 22-VI-2021, 2540 m, Leg.: Z. Nedeljković/DNA CEUA_S84/CEUA00109889; 00109890; 00109898 (CEUA-CIBIO) • 2♀♀—ESPAÑA (GR), Sierra Nevada, Monachil, Sendero al Borreguil de San Juan, 22-VI-2021, 2540 m, Leg.: Z. Nedeljković/DNA CEUA_S87/CEUA00109910; 00109911 (CEUA-CIBIO) • 3♂♂—ESPAÑA (GR), Sierra Nevada, Monachil, Sendero al Borreguil de San Juan, 22-VI-2021, 2540 m, Leg.: P. Aguado Aranda/CEUA00109888; 00109893; 00109894 (CEUA-CIBIO) • 3♂♂—ESPAÑA (GR), Sierra Nevada, Monachil, Sendero al Borreguil de San Juan, 22-VI-2021, 2540 m, Leg.: I. Ballester Torres/DNA CEUA_S161/CEUA00109895; 00109896; 00109897 (CEUA-CIBIO) • 1♂—ESPAÑA (GR), Sierra Nevada, Monachil, Borreguil de San Juan, 22-VI-2021, 2500 m, Leg.: Z. Nedeljković/CEUA00109887 (CEUA-CIBIO) • 2♂♂—ESPAÑA (GR), Sierra Nevada, Monachil, Borreguil de San Juan, 22-VI-2021, 2500 m, Leg.: Mª Á. Marcos García/CEUA00109908; 00109909 (CEUA-CIBIO) • 1♀—ESPAÑA (GR), Sierra Nevada, Monachil, Borreguil de San Juan, 22-VI-2021, 2500 m, Leg.: Mª Á. Marcos García/DNA CEUA_S93/CEUA00109883 (AET) • 1♂—ESPAÑA (GR), Sierra Nevada, Monachil, Padrollano, Estación de esquí, 20-VI-2021, 2180 m, Leg.: Z. Nedeljković/CEUA00109884 (CEUA-CIBIO) • 4♂♂—ESPAÑA (GR), Sierra Nevada, Güejar Sierra, Barranco de San Juan, 27-V-2022, 1400 m, Leg.: Z. Nedeljković/CEUA00111060; 00111061; 00111062; 00111063 (CEUA-CIBIO) • 2♂♂—ESPAÑA (GR), Sierra Nevada, Güejar Sierra, Barranco de San Juan, 27-V-2022, 1400 m, Leg.: P. Aguado/CEUA00111064; 00111065 (CEUA-CIBIO) • 2♂♂—ESPAÑA (GR), Sierra Nevada, Güejar Sierra, Barranco de San Juan, 30-V-2022, 1400 m, Leg.: Z. Nedeljković, I. Ballester & P. Aguado/CEUA00111065; 00111066 (CEUA-CIBIO) • 1♀—ESPAÑA (GR), Sierra Nevada, Güejar Sierra, Barranco de San Juan, 30-V-2022, 1400 m, Leg.: Z. Nedeljković, I. Ballester & P. Aguado/CEUA00111068 (CEUA-CIBIO) • 1♂—P.N. Sierra Nevada, Cabecera río San Juan (Granada); 37.1112 lat -3.3763 long, 2850 m, 19-07-2011 (30S6685903147), Leg: S. Santamaría/*Eumerus sabulonum* (Fallén, 1817), Det.: M.A. Marcos, 2012/CEUA00106009 (CEUA-CIBIO) • 1♂—N. slope Veleta, Sierra Nevada, SPAIN, 3000 m, 20.VII.1960, J.R. Vockeroth {hand written}/CNC Diptera # 156131 (CNC) {as *E. sabulonum*, in [50]}.

Diagnosis. This species can be distinguished by the following combination of characters: bare eyes; in males, eyes not touching along a line (Figure 10A); wide vertical triangle (0.44–0.61 mm) (Figure 10E), posterior surstylar lobe slightly constricted (Figure 11B), interior accessory lobe of the genitalia triangular-shaped (Figure 11B); in females, vertical triangle slightly elevated dorsally in lateral view (Figure 10D), occiput usually with a little orange macula on its dorsal part near the vertex. This species is most similar to *E. sabulonum* (see Taxonomic notes).

Etymology. The specific epithet derives from the Latin ‘petra/ae’ (rock) and refers to the fact that many specimens were collected in rocky habitats, usually resting or thermoregulating on rocks. The specific epithet ‘petrarum’ should be treated as a genitive invariable name.

Description. MALE (holotype). Measurements (mm): *l* = 8.3; *vtw* = 0.6. *Head.* Eyes approximated on a point, narrowly separated each other by a distance equalling the diameter of the frontal ocellus. Eye bare. Face and frontal triangle black, white pollinose and covered with white hairs. Vertical triangle black and with long, erect, white hairs. Ocellar triangle isosceles, with black and white hairs intermixed. Occiput black and purple iridescent (under artificial lighting), covered with hairs shorter than those on the vertical triangle; occiput grey pollinose along the eye margin. Scape and pedicel black; pedicel with white hairs which are longer on the ventral side. Basoflagellomere square (ratio length:width = 1:1), concave ventrally, slightly striated radially and black; basoflagellomere with a round, dark brown fossette at the distal margin. Pedicel and blasoflagellomere sparsely white pollinose (pollinosity more obvious under artificial white lighting). Arista black, bare. *Thorax.* Mesonotum and pleura black. Scutum covered with short, semi-reclined white hairs; scutum with two medial white pollinose vittae slightly exceeding the transverse suture; width of the vittae less than 1/8 of the total width of the scutum. Notopleural sulcus absent. Disc of scutellum with white hairs, with hairs of the same length as those on the scutum; posterior margin of the scutellum with small teeth-like protuberances, each bearing a short white hair apically. Posterior anepisternum, anterior anepimeron and katepisternum on its postero-lateral area with dense, long white hairs. Katatergum with a discrete bunch of white hairs. Pleuron grey pollinose except the posterior anepisternum antero-basally, the katepisternum anterolaterally and the posterior anepimeron. Femora black, apices light brown. Tibiae black, basal third light brown. Pro- and mesobasotarsomeres yellow. Second pro- and mesotarsomeres yellow, black at the base. Third pro- and mesotarsomeres black, ventrally yellow and black at the base. Fourth and fifth pro- and mesotarsomeres black. Metafemur with white hairs, ventrally with hairs of different lengths, including some which are as long as the subapical spinae. Metafemur with an anterior row of nine spinae and a posterior row of six spinae apicoventrally. Metatibia with a basoventral ridge covered with short, reclined and black setae. Metatarsi black; metabasotarsomere and second and third tarsomeres ventrally yellow. Wing membrane extensively microtrichose; posterior margin of the wing with dense, short and brown hairs; margin of ventral calypter with long and yellow hairs; margin of dorsal calypter with hairs shorter than that of ventral calypter; halter dark yellow. *Abdomen.* Terga I and IV black; posterior margin of tergum IV brownish orange. Tergum II orange. Tergum III dark orange. Terga II-IV covered with short, reclined and white hairs (black on middle area of terga II-III); anterior corners of tergum II with long and white hairs. Tergum II with a pair of rounded, white pollinose maculae; maculae on terga III-IV slightly curved. Sterna I-II orange; anterior margin of sternum I black. Sternum III orange brownish. Sternum IV black and flat (without projections); the posterior margin of sternum IV slightly convex. Sterna II-IV with short, semi-reclined white hairs. *Genitalia.* Epandrium with a simple posterior surstylar lobe, spatula-shaped and slightly inclined anteriorly (Figure 11B). Cercus with long hairs. Interior accessory lobe triangular-shaped (Figure 11B). Hypandrium simple, curved almost 90° in the middle (Figure 11A).

FEMALE. Same as males, besides sexual dimorphism, except for the following characters: pollinosity on face mostly present along eye margin; basoflagellomere usually 1.2 wider than long; hairs on scutum slightly shorter.

EGG. Measurements (mm): length = 0.6–0.91 (*n* = 10). White when recently laid but yellowish-white when older. The chorionic sculpture consists of irregularly oval-shaped units with a sinuous margin and punctured surface raised by columns. Units do not touch each other (Figure 12).

Distribution. Sierra Nevada, in Granada province (Figure 7).

Biology. Adults fly from late May to July and can be found in a wide altitudinal range (1400–3000 m) but are mainly associated with high mountain areas (>1900 m). The predominant vegetation in localities at low altitudes was woodlands (*Quercus* sp.) but with grasslands (‘Borreguiles’) at high altitudes.

**Figure 13:**
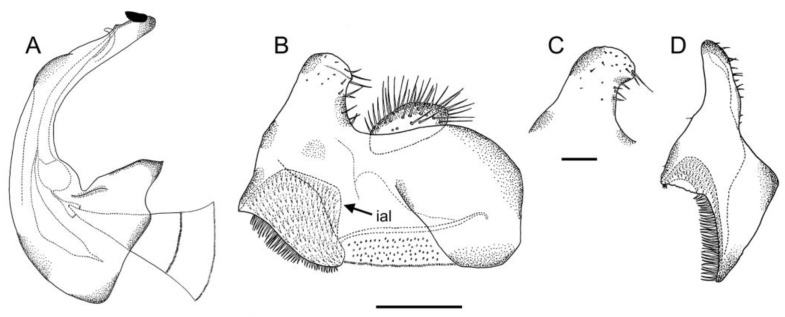
*Eumerus sabulonum*, male genitalia: (**A**) Hypandrium, lateral view (right side). (**B**) Epandrium, lateral view (right side). (**C**) Surstylus, intraspecific variation. (**D**) Epandrium, ventral view. Legend: ial, interior accessory lobe. Scale bars = (**A**,**B**,**D**) 250 µm; (**C**) 100 µm.

Taxonomic notes. *Eumerus petrarum* sp. n. was run through the key of *Eumerus* from Switzerland and surrounding parts of central Europe [30] and the Stackelberg’s key to the Palaearctic *Eumerus* [31], keying out as *E. sabulonum*. The new species differs from *E. sabulonum* in the vertical triangle, which is wider in *E. petrarum* sp. n. (0.44–0.61 mm, *n* = 10) than in *E. sabulonum* (0.33–0.42 mm, *n* = 10); the shape of the posterior surstylar lobe, laterally constricted in *E. petrarum* sp. n. but square-shaped in *E. sabulonum* (Figure 13B); and the interior accessory lobe of the male genitalia, triangular-shaped in *E. petrarum* sp. n. but square in *E. sabulonum* (Figure 13B); in females, occiput with a little orange macula on its dorsal part close to the vertex, which is highly reduced (sometimes absent) in *E. petrarum* sp. n. but usually more noticeable in *E. sabulonum*. Furthermore, based on the examined material, *E. petrarum* sp. n. shows intraspecific variation in the dorsal coloration of protarsomere II (from yellow to black); the coloration of mesotarsomere III (from yellow to black); the number of spinae on the anterior row of the metafemur (six to nine); in males, the morphology of the surstylus (from more to less constricted laterally; Figure 13C); in females, body length is variable (8.04–9.48 mm; *n* = 5). Regarding the chorionic sculpture, the eggs of *E. petrarum* sp. n. are similar to those of *E. sabulonum* but differ on their surface, which is punctured in *E. petrarum* sp. n. (Figure 12) but smooth in *E. sabulonum* (see figure 1 of Munk [51]). In addition, the overall appearance of the eggs of *E. petrarum* sp. n. also resembles those of *Eumerus etnensis* van der Goot, 1964. However, they differ in the shape of the units, which are irregular in *E. petrarum* sp. n., but smooth and rounded with irregular branches in *E. etnensis* (see figure 2 of Pérez-Bañón & Marcos-García [52], as *Eumerus purpurariae* Baéz, 1982).

**Figure 14:**
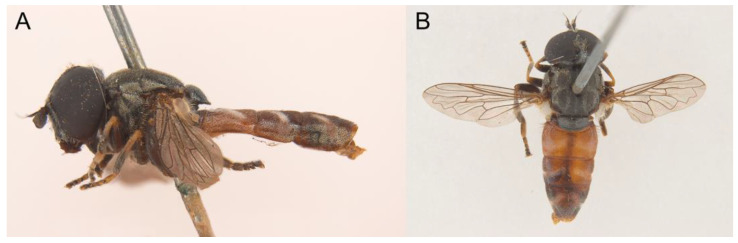
*Eumerus litoralis*, male (syntype), habitus: (**A**) Lateral view. (**B**) Dorsal view. Photos by Simon Hinkley (MMV).

According to Peck [47], three species are listed as synonyms of *E. sabulonum*: *Eumerus litoralis* Curtis, 1839, *Eumerus rubriventris* Macquart, 1829 and *Eumerus selene* Meigen, 1822. The type series of *E. litoralis* consists of five specimens (three males and two females) collected “on the sand hills near Christchurch” in Great Britain. All syntypes are preserved in the MMV collection. We examined pictures of the habitus and the genitalia of one male (Figure 14), which confirmed the synonymy with *E. sabulonum* and its differentiation with the new species. *Eumerus rubriventris* was described based on a single male from the north of France, without a specific locality. According to Horn & Kahle [53], the main section of the Macquart collection was destroyed. The remaining part was divided and preserved between the ‘Museum d’Historie Naturelle’ (MHNL) in Lille, the ‘Museum National d’Historie Naturelle’ (MNHNP) in Paris and the Museum of Natural History of the Oxford University (OUMNH) (Xavier Lair, *in litt.*). There are not records of this species neither in the MNHL (Olivier Boilly, *in litt.*) nor in the MNHPN and the OUMNH. Therefore, we assume that type of material is lost. Moreover, and considering the original (male) description of *E. rubriventris*, the vertical triangle is dark green colored, and the mesonotum exhibits an olive-green coloration, differing from the new species in which both are black. The description of *E. selene* is based on an undetermined number of males from an unknown locality in Europe. The only preserved specimen of this species we have been able to locate is a semi-destroyed female collected in Bavaria, labeled as “*E. silene*, Meig./Allemagne/1155” and deposited in the MNHNP (https://science.mnhn.fr/institution/mnhn/collection/ed/item/ed4105?listIndex=25&listCount=32, accessed on 15 March 2023). Thus, the type series of this species is apparently lost. Regarding the male description of *E. selene*, the white maculae on tergum II are smaller than those on tergum III, differing from the new species in which the maculae of tergum II are the widest of the three pairs.

### 3.3. Remarks on other Iberian Species of the E. tricolor Group

#### 3.3.1. *Eumerus azabense* Ricarte & Marcos–García in Ricarte et al., 2018

**Figure 15:**
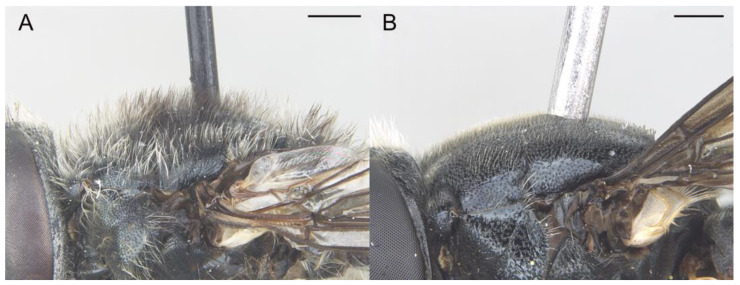
Mesonotum, pilosity (male): (**A**) *E. azabense*. (**B**) *E. tricolor*. Scale bars = (**A**) 750 µm; (**B**) 500 µm.

**Figure 16:**
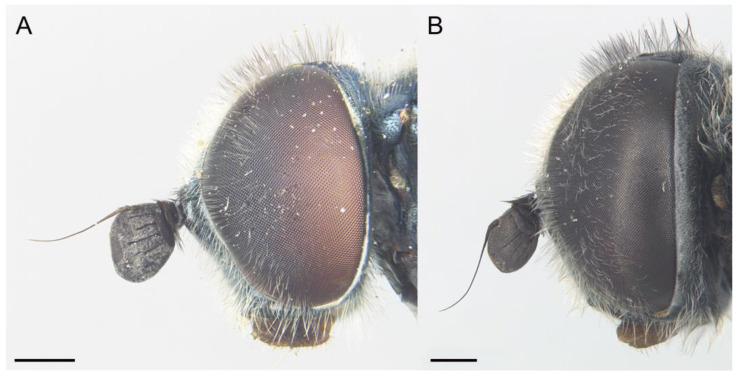
Basoflagellomere, female: (**A**) *E. ovatus*. (**B**) *E. azabense*. Scale bars = 500 µm.

(Figure 15A and Figure 16B)

Distribution. **New.** PORTUGAL • Beja, Almograve. **Published records.** *Revised.* SPAIN • Salamanca, Campanarios de Azaba [14].

#### 3.3.2. *Eumerus bayardi* Séguy, 1961

**Figure 17:**
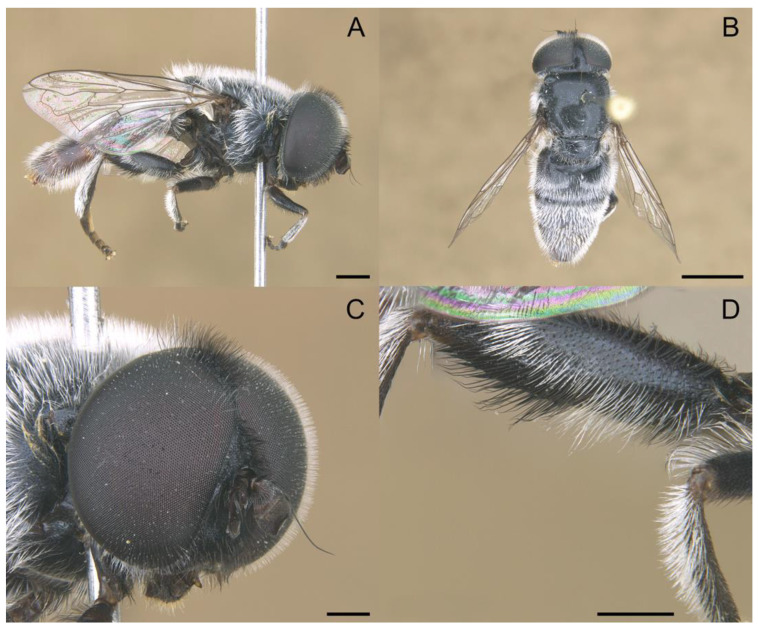
*Eumerus bayardi*, male: (**A**) Habitus, lateral view. (**B**) Habitus, dorsal view. (**C**) Head. (**D**) Metafemur. Scale bars = (**A**) 1 mm; (**B**) 2 mm; (**C**,**D**) 500 µm.

**Figure 18:**
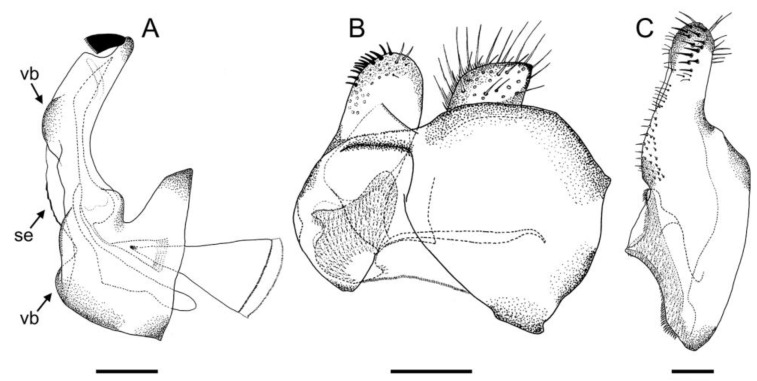
*Eumerus bayardi*, male, genitalia: (**A**) Hypandrium, lateral view (right side). (**B**) Epandrium, lateral view (right side). (**C**) Epandrium, ventral view. Legend: se, serrated expansion; vb, ventral bulge. Scale bars = (**A**,**B**) 250 µm; (**C**) 100 µm.

(Figure 17 and Figure 18)

Distribution. **New.** SPAIN • Almería, Sierra de Gádor. **Published records.** *Revised.* PORTUGAL • Beira Litoral, Serra de Santo António [54].

Genitalia. Epandrium with a simple, rounded posterior surstylar lobe; posterior surstylar lobe with thick, black setae posteriorly; epandrium with a tri-lobed interior accessory lobe (Figure 18B). Cercus rectangular-shaped and slightly pointed anteriorly. Hypandrium with two ventral bulges; hypandrium with an accessory, hyaline ventral expansion, slightly staggered at its basal part (Figure 18A).

Taxonomic notes. This species was described based on a single male⸻from the Jabron Valley (France)⸻of which we examined pictures provided by Dr. Martin Hauser. Speight et al. [55] designated *E. bayardi* as the valid name for *Eumerus micans* (Fabricius, 1798), described originally under the genus *Syrphus* Fabricius, 1775. According to article 57.2 of the International Code of Zoological Nomenclature (ICZN) [56], *E. micans* is a primary homonym of *Syrphus micans* (Fabricius, 1794), which was synonymized with *Volucella inanis* (Linnaeus, 1758) afterward [47]. The name *Eumerus micans* became then invalid, and the species required a new name. Speight et al. [55] indicated that the type material of *E. micans* is lost and that, based on the original description, no differences were found between *E. micans* and *E. bayardi*. Therefore, they synonymized and proposed *E. bayardi* as a replacement name for *E. micans*, following article 60.2 of the ICZN [56].

As a result of our integrative analysis, the status of *E. bayardi* as a member of the *E. tricolor* species group is confirmed. Based on the examined specimens, *E. bayardi* is similar to *E. azabense* but differs in the facial hairs, which are black in *E. bayardi* and white in *E. azabense*; hairs on the scutum, which are entirely white in *E. bayardi* but black on the central area of the scutum in *E. azabense* (Figure 15A); hairs on the basoventral side of the metafemur, white in *E. bayardi* (Figure 17D) and black in *E. azabense*; hairs on the abdomen, white in *E. bayardi* but mainly black in *E. azabense*; and the general shape of the surstylus in male genitalia (for *E. azabense* genitalia, see figure 6b of Ricarte et al. [14]).

#### 3.3.3. *Eumerus grallator* Smit in Grković et al., 2019a

Distribution. **New.** SPAIN • Alicante, Sierra de Aitana; Almería, Sierra de Gádor; Burgos, Los Ausines; Madrid, Las Matas. **Published records.** *Revised.* SPAIN • Alicante, Agres; Valencia, Bocairent [57]. *Not revised.* SPAIN • Burgos, Peñahorada; Ciudad Real, Villahermosa; Granada, Bosque Mediterráneo de Puerto Navazo; Soria, Herrera de Soria [15].

Taxonomic notes. The type series of this species consists of 10 specimens collected in Spain [15]. According to the species diagnosis, one of the characters to distinguish it from other similar species is the relative length of the metatibia, which is clearly shorter than the metafemur. After the examination of further material, we have found that the length of these leg segments is variable amongst individuals (metatibia = 2.37–3.1 mm, metafemur = 2.4–3.1 mm, *n* = 10). The intraspecific variation in the length of these leg segments indicates that this should be avoided as a diagnostic character for species identification.

Furthermore, and based on the original description of the long-legged North African *Eumerus afrarius* Séguy, 1961, the males of *E. grallator* appear to be very similar morphologically to those of *E. afrarius*. Based on the original description of *E. afrarius*, the only differences found between the two species were the eye distance, which is equal to the diameter of an ocellus in *E. grallator* but twice the diameter of an ocellus in *E. afrarius*, and the marginal coloration of the scutellum, which is bluish-black in *E. grallator* but supposedly red in *E. afrarius*. Grković et al. [15] did not mention *E. afrarius* in their revision of the *E. binominatus* subgroup. *Eumerus afrarius* was described from a single male collected in Massena (Algeria), apparently preserved in the MNHNP (http://www.diptera.org/Nomenclator/Details/47859, accessed on 15 May 2023). No records of this species were found in the online database of the MNHNP, nor was information about the holotype available upon request. A potential synonymy between these two species should be further investigated in the future due to their high morphological similarity and the geographical proximity between the Iberian Peninsula and the North of Africa.

#### 3.3.4. *Eumerus grandis* Meigen, 1822

(Figure 4A and Figure 5B)

Distribution. **Published records.** *Not revised.* SPAIN • Girona, Queralbs [11].

Taxonomic notes. The only known Iberian record of this species is a single female collected in the Pyrenees of Girona, near the type locality of *E. ancylostylus* sp. n. Since the female of *E. ancylostylus* sp. n. is unknown and in the absence of molecular data for the above-mentioned female, the record of *E. grandis* from the Pyrenees of Girona is likely to belong to *E. ancylostylus* sp. n. Thus, further fieldwork and molecular analyses are necessary to confirm the presence of *E. grandis* in the Ibero-Balearic area.

#### 3.3.5. *Eumerus hispanicus* van der Goot, 1966

**Figure 19:**
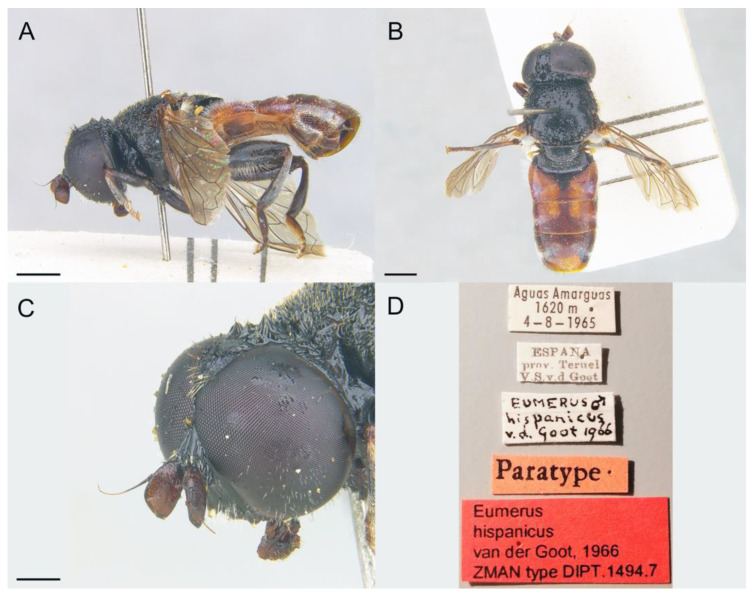
*Eumerus hispanicus*, male (paratype): (**A**) Habitus, lateral view. (**B**) Habitus, dorsal view. (**C**) Head. (**D**) Original labels. Scale bars = (**A**,**B**) 1 mm; (**C**) 500 µm.

**Figure 20:**
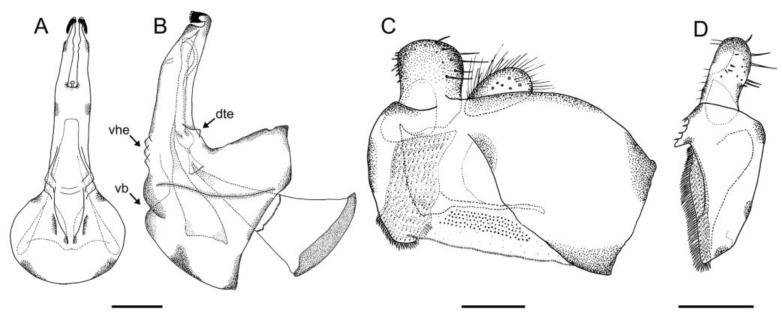
*Eumerus hispanicus*, male genitalia (mainly based on the paratype), hypandrium: (**A**) Ventral view. (**B**) Lateral view (right side). Epandrium: (**C**) Lateral view (right side). (**D**) Ventral view. Legend: dte, dorsal triangular expansion; vb, ventral bulge; vhe, ventral hyaline expansions. Scale bars = 250 µm.

(Figure 19 and Figure 20)

Distribution. **New.** SPAIN • Alicante, Sierra de Aitana, Sierra del Maigmó; Segovia, Maderuelo. **Published records.** *Revised.* SPAIN • Alicante, Agres, Jijona [57]; Teruel, Aguas Amargas [58]; Valencia, Chelva [59].

Genitalia. Epandrium with a simple posterior surstylar lobe, square-shaped and slightly rounded at the top (Figure 20C); epandrium with a rectangular-shaped, black pilose interior accessory lobe of the surstylus (Figure 20C,D). Cercus with long pilosity. Hypandrium with two round bulges basally (Figure 20B); hypandrium with three pointed, hyaline expansions basoventrally (Figure 20A,B); hypandrium with a small, triangular expansion basodorsally (Figure 20B).

#### 3.3.6. *Eumerus larvatus* Aracil, Grković & Pérez-Bañón in Aracil et al., 2023

Distribution. **Published records.** *Not revised.* SPAIN • Alicante, Agost, Albatera; Almería, Sorbas, Tabernas [24].

Taxonomic notes. This species has been recently described from material collected in south-eastern Spain. The type locality is “SPAIN: Agost//Barranc de Pina” [24]. These authors separated males of *E. larvatus* from *Eumerus compertus* Villeneuve in Villeneuve & Gauthier, 1924, *Eumerus mucidus* Bezzi, 1921 and *Eumerus turcmenorum* Paramonov, 1927. Regarding *E. turcmenorum*, the genitalia of *E. larvatus* was compared with those represented in Mutin & Barkalov [60]. However, upon examination of both the genitalia of all other Iberian species of the *E. tricolor* group and photos of the genitalia of a supposed male of *E. turcmenorum* (Martin Hauser, unpublished data), we find the drawing provided by Mutin & Barkalov [60] is inconclusive; these authors appear to draw the posterior surstylar lobe in the cercus’ location, and the structures illustrated in the location of this lobe look ambiguous. In addition, Piwowarczyk & Mielczarek [61], also cited by Aracil et al. [24], provided a picture of the genitalia of *E. compertus,* which in fact, does not belong to *E. compertus* (we have examined a male of *E. compertus* from the CEUA-CIBIO: TUNISIEN, Bou Hedma, 15-17.3.93, leg. & det. Hauser/CEUA00017794). Although the validity of *E. larvatus* is not put in question, new molecular and morphological data should be used to further clarify the systematic and phylogenetic relationships amongst *E. larvatus* and allied species [24].

#### 3.3.7. *Eumerus ovatus* Loew, 1848

(Figure 16A)

Distribution. **Published records**. *Revised.* ANDORRA • Santa Coloma (van Eck & Carles-Tolrá, *in press*). *Not revised.* SPAIN • Girona, Campelles [11], Santa Fe [62], San Marsal [63].

Taxonomic notes. Gil-Collado [63] reported this species from the localities of Santa Fe (Granada) and San Marsal (Girona). The record from the first locality was previously published by Andréu [62] based on a single male but collected close to a marsh located in Santa Fe del Montseny (Girona), not in Granada. In addition, based on the known records, this species is present in rainy, high mountain habitats, which is not the case for the Santa Fe site in Granada, southern Spain. Therefore, the citation of *E. ovatus* from Granada should be a misinterpretation by Gil Collado [63] of the locality data provided by Andréu [62].

#### 3.3.8. *Eumerus sabulonum* (Fallén, 1817)

**Figure 21:**
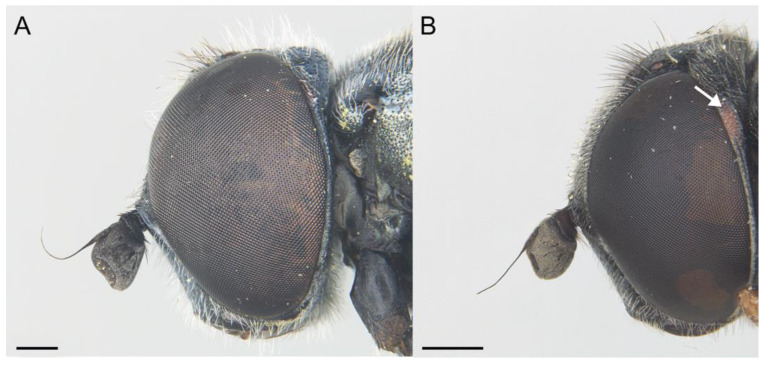
*Eumerus sabulonum*, head, lateral view: (**A**) Male. (**B**) Female. An arrow indicates the orange macula. Scale bars = (**A**) 250 µm; (**B**) 500 µm.

(Figure 13 and Figure 21)

Distribution. **New**. PORTUGAL • Faro, Corte do Gago; Viseu, Póvoa Dão • SPAIN • Ávila, Sierra de Gredos; Granada, Sierra Nevada; Navarra, Refugio de Belagua; Salamanca, Saucelle, Villar de Ciervo; Zamora, San Pedro de Nave. **Published records**. *Revised*. SPAIN • Ávila, Gilbuena; Cáceres, Arrolobos, Puerto de Honduras; Ciudad Real, Parque Nacional de Cabañeros [64]; León, Pereda de Ancares [46]; Madrid, El Ventorrillo [65]; Salamanca, Béjar [66], Navasfrías, Sierra Candelario [67]; Segovia, La Granja de San Ildefonso [66]. *Not revised.* PORTUGAL • Leiria, Citânia de Briteiros; Oporto, Vila do Conde [21] • SPAIN • Cáceres, Hervás; Madrid, El Escorial, Rivas, Sierra de Guadarrama; Pontevedra, La Guardia; Segovia, San Rafael; Vizcaya, Bilbao; Zamora, Bayona [63].

Taxonomic notes. The type series of *E. sabulonum*, described under the genus *Pipiza*, consists of eight specimens, and it is preserved in the entomological collection of the ‘Naturhistoriska riksmuseet’ in Stockholm (catalog numbers NHRS-GULI000070672–79). All these specimens were collected in Sweden. We could not access the type series upon request, but we included in the molecular analyses a barcode sequence of one male specimen (voucher NORSY404-12) collected in Norway, close to the type locality. We considered this specimen as the *E. sabulonum sensu stricto* to compare with those collected in the Iberian area.

#### 3.3.9. *Eumerus tarsalis* Loew, 1848

Distribution. **New**. SPAIN • Granada, Sierra Nevada. **Published records**. *Not revised.* SPAIN • Barcelona [62]; Granada, Sierra Nevada [68]; Madrid, El Escorial [63].

#### 3.3.10. *Eumerus tricolor* (Fabricius, 1798)

(Figure 15B)

Distribution. **New**. SPAIN • Alicante, Alcoleja, Planes; Burgos, Covarrubias; León, Valle de Casares; Madrid, Chinchón, Perales de Tajuña; Santander, Sotres, Vada; Valencia, Bocairent. **Published records**. *Revised.* SPAIN •Alicante, Font Roja [69]; León, Caboalles de Abajo, Mirantes de Luna, Morgovejo [46]; Madrid, Aranjuez, Montarco [63]. *Not revised.* SPAIN • A Coruña, Vilaboa [63].

### 3.4. Key to the European Species of the E. tricolor Group

The key presented below is a result of combining and adapting the key to males of the *E. binominatus* subgroup [15] and the key to the south-eastern European species of the *E. tricolor* group [11]. We decided to exclude *E. lateralis* from the key since its taxonomic status remains uncertain until further molecular evidence is available.

#### Males

1. The segments of the metaleg are remarkably slender (Grković et al. [15]: figure 4) … 2 (*E. binominatus* subgroup)
  − Segments of the metaleg of normal length … 3 (other species)
2. The greatest width of the metafemur is approximately equal to one-fifth of the length of the metafemur (Grković et al. [15]: figure 4) … *E. grallator*
  − The greatest width of the metafemur is approximately equal to one-eighth of the length of the metafemur (Grković et al. [15]: figure 4) … *E. tenuitarsis*
3. The eyes are bare or nearly bare (i.e., with very short and dispersed hairs on the eye surface) … 4
  − The eyes are hairy … 10
4. The basoflagellomere is large, about one-third of the height of the head, and yellow (Grković et al. [11]: figure 4). Tarsomeres are short, and the metabasotarsomere is longer than the other four tarsomeres of the same leg combined (Grković et al. [11]: figure 4). The ocellar triangle is isosceles in nature and closer to eye contiguity than to the upper eye margin … 5
  − The basoflagellomere is small, about one-quarter of the height of the head, and yellow or brown (Grković et al. [11]: figure 6). Tarsi of normal length. The ocellar triangle is wider than it is long or equilateral and closer to the upper corner of the eye than to eye contiguity … 6
5. The hairs on the ventral side of the metafemur are short (Grković et al. [11]: figure 4). The male genitalia, as in figure 5 [11] … *E. armatus*
  − The hairs on the ventral side of the metafemur are very long (Grković et al. [11]: figure 4). The male genitalia, as in figures 5b1–2 [11] … *E. nigrorufus*
6. Basoflagellomere yellow. Tergum I yellow posteriorly (Grković et al. [13]: figure 10) … *E. rubrum*
  − Basoflagellomere black or dark brown. Tergum I is entirely black … 11
7. Hairs on the ventral side of the metafemur are very long and dense and clearly longer than the spinae … *E. tauricus*
  − Hairs on the ventral side of the metafemur are shorter, or only slightly longer, than the spinae … 8
8. The basotarsomere and tarsomeres II–IV of the proleg each bear a long black seta posterolaterally. The male genitalia, as in figure 5d [11] … *E. tarsalis*
  − Protarsus without a long black seta … 9
9. The vertical triangle is wide (Figure 10E). Posterior surstylar lobe constricted (Figure 11B). Interior accessory lobe triangular-shaped (Figure 11B) … *E. petrarum* sp. n.
  − The vertical triangle is narrow. The posterior surstylar lobe is not constricted (Figure 13B). The interior accessory lobe is square-shaped (Figure 13B) … *E. sabulonum*
10. The eyes are separated, at least, by the diameter of an ocellus (Grković et al. [11]: figures 4, 6) … 11
  − The eyes are holoptic or, at most, separated by less than the diameter of an ocellus (Grković et al. [11]: figures 9, 11) … 14
11. The basoflagellomere is yellow or red. The terga are black … 12
  − The basoflagellomere is black or dark brown. The terga have red maculae … 13
12. Tergum IV is covered with short, golden yellow hairs (Grković et al. [11]: figure 6). Posterior surstylar lobe of male genitalia erect (Grković et al. [11]: figure 3) … *E. aurofinis*
  − Tergum IV is not covered with golden yellow hairs. The posterior surstylar lobe of the male genitalia is bent posteriorly (Grković et al. [11]: figure 3) … *E. richteri*
13. The hairs on the mesonotum are very long. Terga II-III have large, black maculae centrally (Grković et al. [11]: figure 11) … *E. sinuatus*
  − The hairs on the mesonotum are short (Figure 15B). Terga II–II are mainly red; tergum II with triangular, black maculae anteromedially (Grković et al. [11]: figure 6) … *E. tricolor*
14. The ventral side of the metafemur is covered with white hairs basally and black hairs distally (Figure 17D) … *E. bayardi*
  − The ventral side of the metafemur is entirely covered with unicolorous hairs… 15
15. The basoflagellomere is square-shaped. Metafemur ventrally covered with long black hairs … *E. larvatus*
  − The basoflagellomere is not square-shaped. Metafemur covered with hairs of different dispositions and/or colours … 16
16. The mesonotum is covered with moderately long hairs (e.g., Figure 3A) … 17
  − The mesonotum is covered with very long hairs (Figure 15A) … 21
17. The terga are entirely black. Posterior surstylar lobe of male genitalia with a distinct lateral wing-like expansion (Grković et al. [11]: figure 3) … 18
  − The terga usually have red maculae laterally. The posterior surstylar lobe of the male genitalia does not have a lateral wing-like protrusion … 19
18. Basoflagellomere rectangular-shaped (Grković et al. [11]: figure 9). The posterior surstylar lobe of male genitalia is short and wide (Grković et al. [11]: figure 3) … *E. crispus*
  − The basoflagellomere is trapezoidal-shaped (Grković et al. [11]: figure 2). The posterior surstylar lobe of male genitalia is long and narrow (Grković et al. [11]: figure 3) … *E. arctus*
19. The ocellar triangle has black and white hairs intermixed. The terga are mainly red … *E. hispanicus*
  − The ocellar triangle has black hairs. The terga are black with red maculae laterally, at least, on tergum II … 20
20. The basoflagellomere is wider than long or, at least, as wide as it is long. The vertical triangle is wide (Figure 4A). The posterior surstylar lobe of the male genitalia is rounded anteriorly (Figure 5A) … *E. ancylostylus* sp. n.
  − The basoflagellomere is longer than it is wide. The vertical triangle is narrow (Figure 4B). The posterior surstylar lobe of the male genitalia is square-shaped (Figure 5B) … *E. grandis*
21. Terga III-IV are covered with characteristic silver-white hairs … *E. ovatus*
  − The hairs on terga II–IV are in a different arrangement … 22
22. The metatibia is covered with characteristic snow-white hairs dorsally (Grković et al. [11]: figure 7). Terga II–IV entirely black with metallic blue shine (Grković et al. [11]: figure 11) … *E. niveitibia*
  − The metatibia is not covered with snow-white hairs. Terga II–IV do not have a metallic blue shine … *E. azabense*

#### Females

1. The metafemur and metatibia are remarkably slender. The spinae on the ventral side of the metafemur are tiny, short and sparse (Grković et al. [11]: figure 8) … 2 (*E. binominatus* subgroup)
  − The metafemur and metatibia are more or less thickened. The spinae on the ventral side of the metafemur are arranged in rows … 3 (other species)
2. The ocellar triangle has white hairs. The sterna have white hairs … *E. grallator*
  − The ocellar triangle has black hairs. The sterna have black hairs except sternum IV, covered with white hairs … *E. tenuitarsis*
3. The eyes are bare or nearly bare … 4
  − The eyes are covered with conspicuous hairs … 9
4. The basoflagellomere is dark brown (Grković et al. [11]: figure 6). Pro- and mesotarsomeres II-III yellow, with a black spot basally (Grković et al. [11]: figure 6) … 5
  − The basoflagellomere is yellow (Grković et al. [11]: figure 4). The pro- and mesotarsus are black … 7
5. Protarsomeres II–IV each have a long black seta posterolaterally … *E. tarsalis*
  − The protarsus does not bear a long black seta … 6
6. The basoflagellomere usually tapers toward the apex. Vertical triangle slightly elevated dorsally (Figure 10D) … *E. petrarum* sp. n.
  − The basoflagellomere is usually axe-shaped. Vertical triangle not elevated dorsally (Figure 21B) … *E. sabulonum*
7. Basoflagellomere greatly enlarged, twice as wide as the pedicel or more (Grković et al. [11]: Figure 4). Metafemur black (Grković et al. [11]: Figure 4) … E. armatus (Greek Islands: Lesvos, Rhodes and Samos) or *E. nigrorufus* (Montenegro)
  − The basoflagellomere is small, about 1.5 times wider than the pedicel. The metafemur is yellow to red (Grković et al. [11]: Figure 8) … 8
8. The ocellar triangle is wider than it is long (Grković et al. [13]: figure 12). The metafemur has a transverse suture anteriorly (Grković et al. [13]: figure 12) … *E. rubrum*
  − The ocellar triangle is equilateral or almost so (Grković et al. [13]: figure 12). The metafemur is without a transverse suture anteriorly … *E. tauricus*
9. Hairs on the mesonotum are short (Figure 15B) or moderately long (e.g., Figure 3A) … 10
  − Hairs on the mesonotum are very long (Figure 15A) … 16
10. The basoflagellomere is yellow or red (Grković et al. [11]: figure 6) … 17
  − The basoflagellomere is black or dark brown … 12
11. The eyes are covered with short, dense white hairs (Grković et al. [11]: figure 4). The metafemur is moderately thickened, covered with dense white hairs (Grković et al. [11]: figure 8e) … *E. richteri*
  − Eyes covered with tiny, scattered hairs (Grković et al. [11]: figure 6). Metafemur strongly thickened and covered with extremely short yellow hairs (Grković et al. [11]: figure 8) … *E. aurofinis*
12. The basoventral hairs of the metafemur are longer than the spinae … 13
  − Basoventral hairs of the metafemur are clearly shorter than the spinae … 15
13. The basoflagellomere is square-shaped … *E. larvatus*
  − The basoflagellomere is rounded … 14
14. The basoflagellomere has a yellow macula basally … *E. grandis*
  − The basoflagellomere is unicolorous … *E. hispanicus*
15. The basoflagellomere is small (Grković et al. [11]: figure 6). The terga are predominately red (Grković et al. [11]: figure 6) … *E. tricolor*
  − The basoflagellomere is big (Grković et al. [11]: figure 9). The terga are predominately black (Grković et al. [11]: figure 9) … *E. crispus*
16. The metatibia is dorsally covered with conspicuous snow-white hairs (Grković et al. [11]: figure 8d). Terga II–III are mainly black, sometimes with reduced red maculae … *E. niveitibia*
  − The metatibia is without conspicuous snow-white hairs. Terga II–III are mainly red … 17
17. The basoflagellomere is oval (Figure 16B) … *E. azabense*
  − The basoflagellomere is rounded (Figure 16A) … 18
18. The basoflagellomere is moderately striated (Grković et al. [11]: figure 11) … *E. ovatus*
  − The basoflagellomere is densely striated (Grković et al. [11]: figure 11) … *E. sinuatus*

## 4. Discussion

After the present work, the number of Iberian species assigned to the *E. tricolor* group grows from nine to twelve (Portugal, three spp.; Spain, twelve spp.; shared species, three), and any of them has been found in the Balearic Islands. In total, six species are endemic to the Iberian Peninsula. This value of alpha diversity is similar to those found for the same species group in other European areas, such as the 13 species reported from the Balkan Peninsula [11]. The taxonomic and systematic research on the *E. tricolor* group is conditioned by the high levels of species richness and phenotypic diversity within some species (such as *E. sabulonum*), as well as the low levels of morphological differentiation for other species (e.g., *E. ancylostylus* sp. n. and *E. grandis*). In fact, there are just a few studies devoted exclusively to the taxonomy and systematics of the *E. tricolor* group in the Palaearctic Region [11,15,16]. The present work is the first in revising the *E. tricolor* group in south-western Europe and in providing a key to all its European species.

The analysis of the COI sequences revealed that *E. petrarum* sp. n. was not only closely related to *E. sabulonum* but also to *E. tarsalis* in a well-supported clade (Figure 2). These species share morphological features such as bare eyes and a small black basoflagellomere, among others. The fact that these sibling lineages share a common ancestor together with low levels of morphological disparity and genetic divergence are clear indicators of a recent divergence event [70]. On the contrary, the resulting trees showed that *E. ancylostylus* sp. n. grouped in a single clade with *E. hispanicus* (Figure 1 and Figure 2). These species can be distinguished by the coloration of terga, mainly red in *E. hispanicus* but black in *E. ancylostylus* sp. n. (small red maculae can appear, at least, on tergum II) and the general shape of male genitalia (Figure 5 and Figure 19). The analysis of COI sequences also suggests that both Iberian species appear to be related to *E. crispus* (Figure 2). This species was recently described from Serbia, in the Balkan Peninsula [11] and differs from the Iberian species in the coloration of terga, which is entirely black in *E. crispus* but with red maculae in *E. ancylostylus* sp. n. and *E. hispanicus*, and a lateral wing-like expansion in the posterior surstylar lobe of male genitalia, which is present in *E. crispus* but absent in *E. ancylostylus* sp. n. and *E. hispanicus*. The discordance between the molecular association of *E. ancylostylus* sp. n. and its phenotypic similarity with *E. grandis* appears to reflect a parallelism occurrence [70]. However, the observed polytomies underlined that the COI gene was not resolutive enough, and additional markers are required to disentangle the evolutionary relationships between these species.

In contrast with their morphological similarity with other species, both new species differ in levels of molecular divergence from their allied ones. On the one hand, *E. ancylostylus* sp. n. showed a 3% of divergence in COI compared to the analyzed European specimens of *E. grandis*. In addition, *E. ancylostylus* sp. n. was revealed to be more closely related to *E. hispanicus* (<1%) than to *E. grandis* (Figure 2). Thus, a speciation process giving rise to the Iberian-endemic lineages of *E. ancylosytus* sp. n. and *E. hispanicus* is plausible. On the other hand, the divergence in COI amongst the individuals of *E. petrarum* sp. n. and *E. sabulonum* was less than 1%. These are not the first reported cases of low genetic divergence between congeneric species in syrphids. For instance, Ricarte et al. [7] highlighted low divergence, in terms of COI-5′, within valid species of the brachyopine genera *Melanogaster* Rondani, 1857 and *Lejogaster* Rondani, 1857. Another example is found in the genus *Merodon,* as low divergence in several molecular markers was also reported within valid species [9]. Nevertheless, our species concepts are well supported by morphological data, confirming the validity of the new species. Therefore, the use of additional sets of data (e.g., genomic, geometric morphometrics), together with the morphology, is required to test the validity of cryptic and/or sibling species.

As a result of this study, the distribution ranges of the Iberian species of the *E. tricolor* group are now better understood (Figure 7). The two new species showed narrow ranges. *Eumerus ancylostylus* sp. n. was recorded from two different localities in the Euro-Siberian region of Spain, on north [26]. Although the two localities are far away from each other, both are montane areas in which the observed vegetation was derived from long periods of human use [44,46]. Similarly, *E. petrarum* sp. n. was collected in a mountainous area (Sierra Nevada) where this species is present from the montane zone to the highest alpine zones. The finding of these undescribed species is an indicator for the Iberian Peninsula to have acted as a climate refuge, in which the mountains were speciation centers for animals and plants during glacial-interglacial events [71,72]. Regarding the other Iberian members of the *E. tricolor* group, *E. sabulonum* was recorded for the first time from the Pyrenees of Navarra, which is the northernmost record in the Iberian territory. This species showed the most cosmopolitan distribution pattern within the Iberian Peninsula since it can be found from meridional areas, almost at sea level, to septentrional mountainous zones. This fact is consistent with the remainder of its distribution range as it is present from North Africa to the European areas of Russia [73]. *Eumerus grallator* was reported from Madrid and Sierra de Gádor (Almería), which is one of the most southern records of this species in the Iberian Peninsula. According to the literature and the examined material, this species is mainly present in montane areas of the Mediterranean Region. On the contrary, based on the Iberian records, *E. ovatus* appears to be associated with humid and rainy habitats, but it can also be found in dry and open environments where herbaceous and shrubby vegetation are predominant [73]. Nevertheless, the low number of records of *E. ovatus*, half of them originating from old publications [11,62,63], may indicate that its Iberian populations are declining like in other European areas [74]. Similarly, the first documented record of *E. bayardi* from Spain is provided on the basis of a single specimen from Almería. The taxonomic status of this species has been uncertain for a long time, as well as its presence in the Ibero-Balearic region [11,18]. Based on the two known records, *E. bayardi* seems to be present in low-temperature Mediterranean areas. After the present work, the morphological concept of this species is clarified, but more fieldwork is necessary in order to fill the gap of knowledge about its distribution range and biology. Moreover, the first record of *E. azabense* from Portugal highlights that incomplete knowledge of species distributions in the Ibero-Balearic area represents a limitation for the conservation of those species that are threatened with extinction [75]. This is particularly alarming for newly discovered species with restricted distributions (e.g., *E. petrarum* sp. n.). Nowadays, 16 European species of the *E. tricolor* group are under one of the IUCN threatened categories, of which five are present in the Iberian Peninsula. Thus, further studies in unexplored areas of the Iberian Peninsula will assist in getting a better picture of the distribution of the species of the *E. tricolor* group in this area and, thus, a more accurate view of their conservation status.

## 5. Conclusions

(1) A total of 12 species of the *E. tricolor* group are present in the Ibero-Balearic region. A lectotype is designated for *E. lateralis*;
(2) Two new species, *E. ancylostylus* sp. n. and *E. petrarum* sp. n., are described, illustrated and discussed;
(3) From a morphological point of view, levels of intraspecific variability were high for both *E. sabulonum* and *E. petrarum* sp. n., while the interspecific variability was low between *E. grandis* and *E. ancylostylus* sp. n.;

This is the first work devoted to improving the knowledge about the diversity, systematics and distribution of the *E. tricolor* species group in the Iberian area.

## Data Availability

All sequences generated in this work are available in the publicly accessible repository of GenBank (https://www.ncbi.nlm.nih.gov/genbank/).

## Supplementary Materials

The following supporting information can be downloaded at: https://www.mdpi.com/article/10.3390/insects14060541/s1, Table S1: List of examined material; Table S2: List of species/specimens analyzed in the molecular study.
