# Supplementary material for: Are Appearances Deceiving? Morpho-Genetic Complexity of the Eumerus tricolor Group (Diptera: Syrphidae) in Europe, with a Focus on the Iberian Peninsula
Source: Insects. 2023 Jun 10;14(6):541. doi: 10.3390/insects14060541 (PMC10299051; doi:10.3390/insects14060541)
Supplement: Supplementary file 1 [file insects-14-00541-s001.zip › Supplementary/Supplementary_S1_Examined_material.pdf]

Eumerus azabense **Ricarte & Marcos-García in Ricarte et al., 2018**

New. PORTUGAL • 1♂ – PT, Almograve, gps 37.6578 -8.8001, 27.III.2023, leg. S. Bot/*Eumerus azabense* Ricarte & Marcos-García, 2018, Det. S. Bot 2023/*Eumerus azabense* Ricarte & Marcos-García in Ricarte et al., 2018, Det. A. Ricarte & P. Aguado (2023) (PSB).

Revised. HOLOTYPE. SPAIN • ♂ – CAMPANARIOS DE AZABA, Salamanca, SPAIN, Malaise 19, 06/05/2011, Leg: Quinto, García, Ramírez/*Eumerus azabense* Ricarte y Marcos García in Ricarte et al. 2018, CEUA A. Ricarte, 2018/HOLOTYPE {red label}/M – ADN, Ante, ex 1 junio 2016, ex = extracción patas {hand written}/CEUA00106286 (CEUA-CIBIO). PARATYPES. SPAIN • 2♂♂ – CAMPANARIOS DE AZABA, Salamanca, SPAIN, Malaise 2, 07/06/2011, Leg: Quinto, García, Ramírez/*Eumerus azabense* Ricarte y Marcos García in Ricarte et al. 2018, CEUA A. Ricarte, 2018/PARATYPE {red label}/CEUA00106339; 00106340 (CEUA-CIBIO) • 1♀ – CAMPANARIOS DE AZABA, Salamanca, SPAIN, Malaise 2, 07/06/2011, Leg: Quinto, García, Ramírez/M – ADN, Ante, ex 1 junio 2016, ex = extracción patas {hand written}/*Eumerus azabense* Ricarte y Marcos García in Ricarte et al. 2018, CEUA A. Ricarte, 2018/PARATYPE {red label}/CEUA00106285 (CEUA-CIBIO) • 1♀ – CAMPANARIOS DE AZABA, Salamanca, SPAIN, Malaise 2, 07/06/2011, Leg: Quinto, García, Ramírez/*Eumerus azabense* Ricarte y Marcos García in Ricarte et al. 2018, CEUA A. Ricarte, 2018/PARATYPE {red label}/CEUA00106338 (CEUA-CIBIO) • 2♀♀ – CAMPANARIOS DE AZABA, Salamanca, SPAIN, Malaise 10, 26/06/2011, Leg: Hernández & Briones/*Eumerus azabense* Ricarte y Marcos García in Ricarte et al. 2018, CEUA A. Ricarte, 2018/PARATYPE {red label}/CEUA00106336; 00106337 (CEUA-CIBIO) • 1♀ – CAMPANARIOS DE AZABA, Salamanca, SPAIN, 26/06/2011, Malaise 19, Leg: Hernández & Briones/*Eumerus azabense* Ricarte y Marcos García in Ricarte et al. 2018, CEUA A. Ricarte, 2018/PARATYPE {red label}/CEUA00106334 (CEUA-CIBIO) • 1♀ – CAMPANARIOS DE AZABA, Salamanca, SPAIN, 26/06/2011, Malaise 20, Leg: Hernández & Briones/*Eumerus azabense* Ricarte y Marcos García in Ricarte et al. 2018, CEUA A. Ricarte, 2018/PARATYPE {red label}/CEUA00106335 (CEUA-CIBIO).

Eumerus bayardi **Séguy, 1961**

New. SPAIN • 1♂ – SPAIN; ALMERIA, Sierra Gádor, 36°49′20.0′′N – 2°43′16.6′′W, 01.V.2014, A. Gonçalves {hand written}/*Eumerus bayardi* Seguy, ♂, det. A. v. Eck, 2015 {species name hand written}/coll. A. v. Eck {yellow label}/ZFMK DIP 00055339/CEUA_S231 (AET).

Revised. PORTUGAL • 1♂ – PORTUGAL; Leiria, Serra de Sto António, 39.526°N 8.743°W, 11.X.2018, A. v. Eck. {leg}/Killed in 96% ethanol {hand written}/*Eumerus bayardi* Séguy, ♂, det. A. v. Eck, 2019 {species name hand

written}/coll. A. v. Eck/ZFMK DIP 00055327/CEUA_S232 (AET) • 1 ♂ – PORTUGAL; Leiria, Serra de Sto António, 39.526°N 8.743°W, 12.X.2018, A. v. Eck. {leg}/*Eumerus bayardi* Séguy, ♂, det. A. v. Eck, 2019 {species name hand written}/coll. A. v. Eck/ZFMK DIP 00055333 (AET) {published in [54]}.

***Eumerus grallator* Smit in Grković et al., 2019a**

New. SPAIN • 5♂♂ – ESPAÑA, Alicante, Sierra de Aitana, Mas del Piscul, campo de trigo, 20.VI.2016, leg. G.J. Souba Dols/*Eumerus grallator* Smit, 2019, Det. Z. Nedeljković & A. Ricarte/CEUA00110320; *Eumerus grallator* Smit, 2019, Det. Ricarte & Nedeljković/CEUA00110321; 0110322; 00110324; 00110326 (CEUA-CIBIO) • 7♂♂ – ESPAÑA, Alicante, Sierra de Aitana, Mas del Piscul (Punto 3), 20.VI.2016, leg. A. Ricarte/*Eumerus grallator* Smit, 2019, Det. Z. Nedeljković & A. Ricarte/CEUA00110319; *Eumerus grallator* Smit, 2019, Det. Ricarte & Nedeljković/CEUA00110323; 00110325; 00110327; 00110328; 00110329; 00110330 (CEUA-CIBIO) • 3♂♂ – ESPAÑA, Alicante, Alcoleja, Sierra Aitana, puerto de Tudons, campo de *Thapsia villosa*, 02-VII-2020, Leg.: Z. Nedeljković / FAUNA IBERICA, ERISTALINAE/*Eumerus grallator* Smit, 2019, Det. Z. Nedeljković & A. Ricarte/DNA CEUA_S70/CEUA00110331; DNA CEUA_S71/00110332; 00110335 (CEUA-CIBIO) • 2♂♂ – ESPAÑA, Alicante, Alcoleja, Sierra Aitana, puerto de Tudons, campo de *Thapsia villosa*, 02-VII-2020, Leg.: A. Ricarte/FAUNA IBERICA, ERISTALINAE/*Eumerus grallator* Smit, 2019, Det. Z. Nedeljković & A. Ricarte/CEUA00110334; 00110336 (CEUA-CIBIO) • 1♂ – ESPAÑA, Alicante, Alcoleja, Sierra Aitana, puerto de Tudons, campo de *Thapsia villosa*, 18-VI-2020, Leg.: Z. Nedeljković/FAUNA IBERICA, ERISTALINAE/*Eumerus grallator* Smit, 2019, Det. Ricarte & Nedeljković/CEUA00110318 (CEUA-CIBIO) • 1♂ – ESPAÑA, Alicante, Alcoleja, Sierra Aitana, puerto de Tudons, campo de *Thapsia villosa*, 18-VI-2020, Leg.: A. Ricarte/*Eumerus grallator* Smit, 2019, Det. Z. Nedeljković & A. Ricarte/CEUA00110333 (CEUA-CIBIO) • 1♀ – ESPAÑA, Alicante, Alcoleja, Sierra Aitana, puerto de Tudons, campo de *Thapsia villosa*, 18-VI-2020, Leg.: A. Ricarte/FAUNA IBERICA, ERISTALINAE/*Eumerus grallator* Smit, 2019, Det. Ricarte & Nedeljković/DNA CEUA_S72/CEUA00110339 (CEUA-CIBIO) • 1♂ – ESPAÑA, Alicante, Alcoleja, Sierra Aitana, puerto de Tudons, campo de *Thapsia villosa*, 20-VI-2020, Leg.: A. Ricarte/FAUNA IBERICA, ERISTALINAE/*Eumerus grallator* Smit, 2019, Det. Z. Nedeljković & A. Ricarte/CEUA00110317 (CEUA-CIBIO) • 1♂ – ESPAÑA, Valencia, Alt del Portell, Bocairent, Vall ´Albaida, 837 m, 12-VI-2021, Leg.: A. Ricarte/CEUA00113569 (CEUA-CIBIO) • 1♂ – ESPAÑA, Almería, Sª de Gádor, Huécija, Camino Cerro de la Cruz, camino a la Ermita, 25-V-2022, Leg.: P. Aguado/CEUA00113570 (CEUA-CIBIO) • 1♂ – ESPAÑA, Almería, Sª de Gádor, Huécija, Camino Cerro de la Cruz, camino a la Ermita, 25-V-2022, Leg.: Z. Nedeljković/CEUA00113571 (CEUA-CIBIO) • 1♂ – SPAIN, Madrid, Las Matas, +755m, UTM: 30T 422 4489, 18.VI.2016, Leg.: P.A. Fidalgo/*Eumerus grallator* Smit, Det. A. v. Eck, 2022 (AET)

• 1♂ – SPAIN, Burgos, Los Ausines, pan trap, UTM: 30T 449.26 4674.41, +908m, 5.VII.2017, Leg.: P.A. Fidalgo/*Eumerus grallator* Smit, Det. A. v. Eck, 2023 (AET).

Revised. SPAIN • 1♂ – Mas del Parral, 900 m, Bocairent (VALENCIA), 3-17/VII/01 {Malaise trap}, Leg.: Pérez Bañón, Marcos-García y Rojo/*Eumerus afrarius* Séguy, 1961, Det.: A. Ricarte, 2005/*Eumerus grallator* Smit, 2019, Det. P. Aguado-Aranda, 2021/CEUA00110337 (CEUA-CIBIO) {published in [57]} • 1♂ – Mas del Parral, 900 m, Bocairent (VALENCIA), 16-30/VII/02 {Malaise trap}, Leg.: Pérez Bañón, Marcos-García y Rojo/*Eumerus grallator* Smit, 2019, Det. P. Aguado-Aranda, 2021/CEUA00110338 (CEUA-CIBIO) {published in [57]} • 1♀ – Caveta del Voltor, 1200 m, Agres (ALICANTE), 17/VI-2/VII/02 {Malaise trap}, Leg.: Pérez Bañón, Marcos-García y Rojo/*Eumerus afrarius* Séguy, 1961, Det.: A. Ricarte, 2005/CEUA00113485 (CEUA-CIBIO) {published in [57]} • 4♀♀ – Foia Ampla, 1060 m, Agres (ALICANTE), 2-16/VII/02 {Malaise trap}, Leg.: Pérez Bañón, Marcos-García y Rojo {identified as *Eumerus afrarius* Séguy, 1961 by X. Mengual}/CEUA00113486; 00113487; 00113488; 00113489 (CEUA-CIBIO) {published in [57]} • 2♀♀ – Foia Ampla, 1060 m, Agres (ALICANTE), 19/VI-3/VII/01 {Malaise trap}, Leg.: Pérez Bañón, Marcos-García y Rojo {identified as *Eumerus afrarius* Séguy, 1961 by X. Mengual}/CEUA00113492; 00113493 (CEUA-CIBIO) {published in [57]} • 2♀♀ – Mas del Parral, 900 m, Bocairent (Valencia), 5-19/VI/01 {Malaise trap}, Leg.: Pérez Bañón, Marcos-García y Rojo {identified as *Eumerus afrarius* Séguy, 1961 by X. Mengual}/CEUA00113490; 00113491 (CEUA-CIBIO) {published in [57]}.

Eumerus grandis **Meigen, 1822**

New. SLOVENIA • 2♂♂ – Slovenia: NW of Nanos, 45°47′10′′N 14 ° 2′11′′E, 24.vi.2015, {leg} J.H., A.M. and A.W. Skevington, M. de Groot, CNC437681; 437772/*Eumerus*, Det. A.D. Young, 2016 (CNC).

Revised. MONTENEGRO • 1♂ – Durmitor, 6.7.1994, Kanjon Sušice, YU, leg. Vujić/*Eumerus grandis* Mg., det. Vujić/CEUA00113476 (CEUA-CIBIO) • POLAND • 1♀ – Polonia mer., Mts. Pieniny, Nowa Góra, 15.VI.1961, leg. K. Malski/*Eumerus annulatus* Panz. {hand written}/*Eumerus grandis* Meigen, 1822, Det: M.A. Marcos-García/CEUA00017801 (CEUA-CIBIO).

Eumerus hispanicus **van der Goot, 1966**

New. SPAIN • 1♂ – ESPAÑA, Alicante, Alcoleja, Sierra Aitana, Puerto de Tudons, campo de *Thapsia villosa*, 02-VII-2020, Leg.: A. Ricarte/FAUNA IBERICA, ERISTALINAE/DNA CEUA_S66/CEUA00113567 (CEUA-CIBIO) • 2♂♂ – ESPAÑA, Alicante, Tibi, Sª del Maigmó, Balcón de Alicante, 22-V-2020, Leg.: A. Ricarte, Z. Nedeljković & M.A Marcos/FAUNA IBERICA, ERISTALINAE/CEUA00113564; 00113565 (CEUA-CIBIO) • 1♀ – ESPAÑA, Alicante, Tibi, Sª del Maigmó, Balcón de Alicante, 22-V-2020, Leg.: A. Ricarte, Z. Nedeljković & M.A Marcos/FAUNA IBERICA, ERISTALINAE/*Eumerus*

hispanicus van der Goot, 1966, Det. Nedeljković & Ricarte/DNA CEUA_S67/CEUA00113566 (CEUA-CIBIO).

Revised. PARATYPE. SPAIN • ♂ – Aguas Amarguas, 1620 m, 4-8-1965 / ESPANA, prov. Teruel, V.S. v.d Goot/EUMERUS ♂ hispanicus, v.d. Goot 1966 {hand written}/Paratype {orange label}/*Eumerus hispanicus* van der Goot, 1966, ZMAN type DIPT.1494.7 {red label} (NBC). SPAIN • 1♂ – Chelva (VALENCIA), 27-VIII-11-IX-94 {Malaise trap}, Leg.: C. Pérez-Bañón/*Eumerus hispanicus* van der Goot, 1966, Det: M.A. Marcos-García/CEUA00017802 (CEUA-CIBIO) {published in [59]} • 1♀ – Chelva (VALENCIA), 24-V-7-VI-94 {Malaise trap}, Leg.: C. Pérez-Bañón/*Eumerus hispanicus* van der Goot, 1966, Det: M.A. Marcos-García/CEUA00017803 (CEUA-CIBIO) {published in [59]} • 1♀ – Caveta del Voltor, 1200 m., Agres (ALICANTE), 2-16/VII/02 {Malaise trap}, Leg.: Pérez-Bañón, Marcos-García y Rojo/*Eumerus hispanicus* van der Goot, 1966, Det.: A. Ricarte, 2005/CEUA00113479 (CEUA-CIBIO) {published in [57]} • 2♀♀ – Caveta del Voltor, 1200 m., Agres (ALICANTE), 2-16/VII/02 {Malaise trap}, Leg.: Pérez-Bañón, Marcos-García y Rojo/CEUA00113481; 00113482 {published in [57]} • 1♀ – Caveta del Voltor, 1200 m., Agres (ALICANTE), 4-17/VI/02 {Malaise trap}, Leg.: Pérez-Bañón, Marcos-García y Rojo/CEUA00113483 (CEUA-CIBIO) {published in [57]} • 1♀ – Font Retura, 900 m., Agres (ALICANTE), 3-17/VII/01 {Malaise trap}, Leg.: Pérez-Bañón, Marcos-García y Rojo/CEUA00113484 (CEUA-CIBIO) {published in [57]} • 2♂♂ – Venta de la Carrasqueta, 980 m., Jijona (ALICANTE), 16-30/VIII/02 {Malaise trap}, Leg.: Pérez-Bañón, Marcos-García y Rojo/CEUA00113477; 00113478 (CEUA-CIBIO) {published in [57]} • 1♂ – Caveta del Voltor, 1200 m., Agres (ALICANTE), 17/VI-2/VII/02 {Malaise trap}, Leg.: Pérez-Bañón, Marcos-García y Rojo/CEUA00113568 (CEUA-CIBIO) {published in Mengual [57]} • 1♀ – Maderuelo (Segovia), 4-VII-2011, Platos, F. Fresno leg./*Eumerus hispanicus* van der Goot, 1966, Det. P. Aguado Aranda, 2022/MNCN_Ent105173 (MNCN).

***Eumerus lateralis* (Zetterstedt, 1819)**

Revised. LECTOTYPE (here designated). SWEDEN • ♂ – E. lateralis ♂, Öland {hand written}/Photo 2022 by MZLU/MZLU 00179349 {yellow label} /MZLU 2022 150 {green label} (MZLU) • PARALECTOTYPE. SWEDEN • ♀ – E. lateralis ♀, Öland {hand written}/Photo 2022 by MZLU/MZLU 00179350 {yellow label}/MZLU 2022 151 {green label} (MZLU).

***Eumerus niveitibia* Becker, 1921**

New. GREECE • 2♂♂ – Greece: Lesbos, SE of Agiasos, 39.0548°N 26.3974°E, 8.ix.2019, {leg} J.H. Skevington, CNC1583962; 1583963/*Eumerus niveitibia* {hand written}, Det. J.H. Skevington, 2019/*Eumerus niveitibia* Becker, 1921, Det. P. Aguado, 2022 (CNC).

***Eumerus ovatus* Loew, 1848**

Revised. 1♂ – 4651/2/g4 {green label}/Zeg 24 8 {no further information about locality; all labels hand written} / *Eumerus ovatus* Loew./CEUA00017687 (CEUA-CIBIO) • ANDORRA • 1♀ – agosto 1992, Santa Coloma (Andorra), Malaise, J. Pujade leg./*Eumerus ovatus* Loew ♀ {hand written}, det. A. van Eck, 2009/coll. A. v. Eck (AET) {(van Eck & Carles-Tolrá, *in press*)} • POLAND • 1 ♀ Toruń wydmy {Poland}, 5.VII.72, Szadziouski leg./*Eumerus annulatus* Panzer /CEUA00017800 (CEUA-CIBIO).

Eumerus sabulonum **(Fallén, 1817)**

New. SPAIN • 3♂♂ – ESPAÑA (GR), Sierra Nevada, Güejar Sierra, Barranco de San Juan, 23-VI-2021, 1400 m, Leg.: Z. Nedeljković/DNA CEUA_S159/CEUA00109871; DNA CEUA_S162/00109872; 00109874 (CEUA-CIBIO) • 2♂♂ – ESPAÑA (GR), Sierra Nevada, Güejar Sierra, Barranco de San Juan, 23-VI-2021, 1400 m, Leg.: P. Aguado Aranda/DNA CEUA_S81/CEUA00109870; 00109873 (CEUA-CIBIO) • 7♂♂ – ESPAÑA (GR), Sierra Nevada, Güejar Sierra, Barranco de San Juan, 27-V-2022, 1400 m, Leg.: Z. Nedeljković/CEUA00111051; 00111052; 00111053; 00111054; 00111055; DNA CEUA_S207/00111056; DNA CEUA_S196/00111057 (CEUA-CIBIO) • 3♂♂ – ESPAÑA (GR), Sierra Nevada, Güejar Sierra, Barranco de San Juan, 27-V-2022, 1400 m, Leg.: P. Aguado/CEUA00111048; 00111049; 00111050 (CEUA-CIBIO) • 1♂ – ESPAÑA (GR), Sierra Nevada, Güejar Sierra, Barranco de San Juan, 27-V-2022, 1400 m, Leg.: I. Ballester/CEUA00111058 (CEUA-CIBIO) • 1♂ – ESPAÑA (GR), Sierra Nevada, Güejar Sierra, Barranco de San Juan, 27-V-2022, 1400 m, Leg.: Z. Nedeljković, I. Ballester & P. Aguado/CEUA00111059 (CEUA-CIBIO) • 1♂ – ESPAÑA (GR), Sierra Nevada, Monachil, Pradollano, Estación de esquí, 20-VI-2021, 2180 m, Leg.: P. Aguado Aranda/CEUA00109882 (CEUA-CIBIO) • 1♀ – ESPAÑA (GR), Sierra Nevada, Güejar Sierra, El Dornajo, Parking camino a Peña del Perro, 24-VI-2021, 1895 m, Leg.: Mª Á. Marcos García/'En flores de' {On flowers of} *Euphorbia nicaeensis* All./DNA CEUA_S82/CEUA00109875 (CEUA-CIBIO) • 3♂♂ – ESPAÑA, Navarra, Refugio de Belagua, 1422 m, 19-VII-2022, Leg.: Antonio Ricarte/CEUA00113470; 00113471; 00113472 (CEUA-CIBIO) • 2♂♂ – ESPAÑA, Navarra, Refugio de Belagua, 1422 m, 19-VII-2022, Leg.: Zorica Nedeljković/CEUA00113474; 00113475 (CEUA-CIBIO) • 1 ♂ – Saucelle (SA), 24-IV-77, Leg.: G. Llorente/*Eumerus sabulonum* (Fallén), Det.: Mª A. Marcos-García/CEUA00017830 (CEUA-CIBIO) • 2♂♂ – SPAIN: Salamanca, Villar de Ciervo, 40.73217°N 6.73223°W, 8-14.iv.2001, H.P. Tschorsnig, YPT/*Eumerus sabulonum* (Fallén) ♂ {hand written}, det. A. van Eck, 2009/coll. A. v. Eck (AET) • 1♀ – SPAIN: Salamanca, Villar de Ciervo, 40.73217°N 6.73223°W, 8-14.iv.2001, H.P. Tschorsnig, YPT/*Eumerus sabulonum* (Fallén) ♀ {hand written}, det. A. van Eck, 2009/coll. A. v. Eck (AET) • 1♀ – España (Zamora), San Pedro de Nave, N 41°34′49′′ W 5°58′45′′, Oever stuwmeer {shore of the reservoir}, *21–V–2019*, 686 m, leg. Wim Klein/*Eumerus tarsalis* (Fallén) ♀ {hand written}, det. A. van Eck, 2020/coll. A. v. Eck (AET) • PORTUGAL • 1♂ – PORTUGAL; Faro Corte do

Gago, 37.2986°N 7.5539°W, 14.IV.2017, V. Jacinto/*Eumerus sabulonum* (Fallén) ♂ {hand written}, det. A. van Eck, 2020/coll. A. v. Eck (AET) • 1♀ – PORTUGAL, Viseu, Silgueiros, Póvoa Dão, UTM 29T589-4489, 15.VI.2013, Almeida leg. {hand written}/2013.VI.15, Póvoa Dão/*Eumerus sabulonum* (Fallén) ♀ {hand written}, det. A. van Eck, 2014/coll. A. v. Eck (AET).

Revised. SPAIN • 1♂ – Pereda de Ancares – LEÓN, 4-VII-87, Mª A. Marcos-García {leg}/*Eumerus sabulonum*, Det: M.A. Marcos/CEUA00017836 (CEUA-CIBIO) {published in [45]} • 1♂ – Pereda de Ancares – LEÓN, 1-VI-87, Mª A. Marcos-García {leg}/*Eumerus sabulonum* (Fallén), Det.: Mª A. Marcos-García/CEUA00017837 (CEUA-CIBIO) {published in [45]} • 1♀ – Pereda de Ancares – LEÓN, 1-VI-87, Mª A. Marcos-García {leg}/*Eumerus sabulonum* (Fallén), Det.: Mª A. Marcos-García/CEUA00017896 (CEUA-CIBIO) {published in [45]} • 3♂♂ – Sierra Candelario (AV) {Salamanca}, 2300 m, 14-VII-80, Leg. Mª A. Marcos/*Eumerus sabulonum* (Fallén), Det.: Mª A. Marcos-García/CEUA00017833; 00017834; 00017890 (CEUA-CIBIO) {published in [67]} • 4♀♀ – Sierra Candelario (AV) {Salamanca}, 2300 m, 14-VII-80, Leg. Mª A. Marcos/*Eumerus sabulonum* (Fallén), Det.: Mª A. Marcos-García/CEUA00017891; 00017892; 00017893; 00017894 (CEUA-CIBIO) {published in [67]} • 1♂ – Sierra Candelario, río (SA), 2100 m, 14-VII-80, Leg. Mª A. Marcos/*Eumerus sabulonum* (Fallén), Det.: Mª A. Marcos-García/CEUA00017835 (CEUA-CIBIO) {published in [67]} • 1♀ – Sierra Candelario, río (SA), 2100 m, 14-VII-80, Leg. Mª A. Marcos/*Eumerus sabulonum* (Fallén), Det.: Mª A. Marcos-García/CEUA00017895 (CEUA-CIBIO) {published in [67]} • 1♂ – Sierra Candelario (AV) {Salamanca}, 2200 m, en laguna mediana, 14-VII-80, Leg. Mª A. Marcos/*Eumerus sabulonum* (Fallén), Det.: Mª A. Marcos-García/CEUA00017832 (CEUA-CIBIO) {published in [67]} • 1♂ – P^{to} Honduras (CC), 1400 m, 17-V-80, Leg. Mª A. Marcos/*Eumerus sabulonum* (Fallén), Det.: Mª A. Marcos-García/CEUA00017831 (CEUA-CIBIO) {published in [67]} • 1♂ – Arrolobos (CC), 1400 m, 4-VI-80, Leg. Mª A. Marcos/*Eumerus sabulonum* (Fallén), Det.: Mª A. Marcos-García/CEUA00017839 (CEUA-CIBIO) {published in [67]} • 1♀ – Navasfrías (SA), 7-VI-1982, en *Anthemis mixta*, Leg. Mª A. Marcos/*Eumerus sabulonum* (Fallén), Det.: Mª A. Marcos-García/CEUA00017898 (CEUA-CIBIO) {published in [67]} • 1♀ – Gilbuena (AV), 28-V-78, 1120 m, S. Fdez. Gayubo {leg} /*Eumerus sabulonum* (Fallén), Det.: Mª A. Marcos-García /CEUA00017897 (CEUA-CIBIO) {published in [67]} • 1♂ – El Ventorrillo, Madrid, ESPAÑA, 1480 m, Tr. Malaise, 9-16/VI-1989, Nieves & Rey leg./*Eumerus sabulonum* (Fallén, 1817), Det: A. Ricarte & D. Lorenzo/CEUA00107523 (CEUA-CIBIO) {published in [65]} • 1♀ – El Ventorrillo, Madrid, ESPAÑA, 1480 m, Tr. Malaise, 9-16/VI-1989, Nieves & Rey leg./*Eumerus sabulonum* (Fallén, 1817), Det: A. Ricarte & D. Lorenzo/CEUA00107523 (CEUA-CIBIO) {published in [65]} • 1♂ – España, Ciudad Real, P.N. de Cabañeros, maM1 {Malaise trap}, 8/30-V-2004, Leg.: A. Ricarte/*Eumerus sabulonum* (Fallén, 1817), Det.: A. Ricarte, 2006/CEUA00084821 (CEUA-CIBIO) {published in [64]} • 4♂♂ – España, Ciudad Real, P.N. de

Cabañeros, maM1 {Malaise trap}, 30-V/18-VI-2004, Leg.: A. Ricarte/*Eumerus sabulonum* (Fallén, 1817), Det.: A. Ricarte, 2006/CEUA00084822; 00084823; 00084824; 00084825 (CEUA-CIBIO) {published in [64]} • 1♂ – España, Ciudad Real, P.N. de Cabañeros, maM2 {Malaise trap}, 30-V/18-VI-2004, Leg.: A. Ricarte/*Eumerus sabulonum* (Fallén, 1817), Det.: A. Ricarte, 2006/CEUA00084826 (CEUA-CIBIO) {published in [64]} • 1♂ – España, Ciudad Real, P.N. de Cabañeros, Pa2 {sampling point}, 21-VI-2004, Leg.: A. Ricarte/*Eumerus sabulonum* (Fallén, 1817), Det.: A. Ricarte, 2006/CEUA00084820 (CEUA-CIBIO) {published in [64]} • 1♀ – España, Ciudad Real, P.N. de Cabañeros, maF1 {Malaise trap}, 29-V/17-VI-2004, Leg.: A. Ricarte/*Eumerus sabulonum* (Fallén, 1817), Det.: A. Ricarte, 2005/CEUA00084827 (CEUA-CIBIO) {published in [64]} • 1♂ – Escorial, Dusmet {leg}/*Eumerus sabulonum* (Fallén, 1817), Det. P. Aguado Aranda, 2022/MNCN_Ent301599 (MNCN) • 1♂ – SPAIN: SALAMANCA, Bejar, Rio Cuerpo de Hombre, 40°23′N 5°45′W, 13.VI.2001, A. v. Eck/*Eumerus sabulonum* (Fallén) ♂, det. A. Vujić, 2008 {hand written}/coll. A. v. Eck (AET) {published in [66]} • 1♂ – SPAIN: SEGOVIA, La Granja, 4°00′ - 40°54′05′′, 08.VI.2001, A. v. Eck/*Eumerus sabulonum* (Fallén) ♂, det. A. Vujić, 2008 {hand written}/coll. A. v. Eck (AET) {published in [66]} • 1♂ – ESPANA, Avila, V.S.v.d. Goot J.A.W. Lucas/Sierra de Gredos, 12 km SSW, Hoyos del Espino, 1950 – 2100 m. 4 - VII - 1972/*Eumerus tarsalis* Loew, det J.A.W. Lucas {hand written} (PJSA) • 1♂ – ESPANA, Avila, V.S.v.d. Goot J.A.W. Lucas/Sierra de Gredos, Navarredonda de G., 1600 – 1700 m. 2 - VII - 1972/*Eumerus tarsalis* Loew, det J.A.W. Lucas {hand written} (PJSA).

***Eumerus tarsalis* Loew, 1848**

New. SPAIN • 2♂♂ – ESPAÑA (GR), Sierra Nevada, Monachil, Sendero al Borreguil de San Juan, 22-VI-2021, 2540 m, Leg.: I. Ballester Torres/CEUA00109877; 00109878 (CEUA-CIBIO) • 1♂ – ESPAÑA (GR), Sierra Nevada, Monachil, Sendero al Borreguil de San Juan, 22-VI-2021, 2540 m, Leg.: A. Ricarte/CEUA00109879 (CEUA-CIBIO) • 1♂ – ESPAÑA (GR), Sierra Nevada, Monachil, Borreguil de San Juan, 22-VI-2021, 2500 m, Leg.: Mª Á. Marcos García/DNA CEUA_S92/CEUA00109880 (CEUA-CIBIO) • 1♂ – ESPAÑA (GR), Sierra Nevada, Güejar Sierra, El Dornajo, Camino a Peña del Perro, 21-VI-2021, 1855 m, Leg.: A. Ricarte/CEUA00109881 (CEUA-CIBIO).

Revised. SPAIN • 1♂ – 37° 7.33′ 3° 25.86′, 2099 m, Ahí de Cara, Sª Nevada (Granada, E), 16-6-2005, Leg.: J. Bosch/'Fuera censo', Em24 {hand written}/*Eumerus sabulonum* (Fallén, 1817), Det.: A. Ricarte, 2006/*Eumerus tarsalis*, det. M. Speight, 11/CEUA00017838 (CEUA-CIBIO) • FRANCE • 1♂ – FRANKRUK, Dept. Hautes Alpes, W. + J. van Steenis/LES VIGNAEAUX, weitje 2 km. Z. 1100-1300m., 8 - vi – 1992 / Eumerus tarsalis Loew, 1848, det. J. van Steenis 1998 (PJSA) • 1♀ – FRANCE – Hautes Alpes, Les Vigneaux, 44° 49′25′′ N 6° 31′ 39′′ E, 4 - VI – 2010, alt. 1100m, W. van Steenis (PJSA) • 1♀ – France, Hautes-

Alpes, Ailefroide camping, 44°52′40′′ N 6°26′00′′ E, 22-VII-2010, 1600 m.a.s.l., J. van Steenis/CEUA00112478 (CEUA-CIBIO) • SWITZERLAND • 1♂ – SCHWEIZ: Wallis Oberwald. Gommer Höhenweg. 1368-1550m, 8.VI.1993, Leg. J.A.W. Lucas/*Eumerus tarsalis* Loew, det J.A.W. Lucas (PJSA) • 1♀ – SCHWEIZ: Wallis, Martigny-Croix, 470m, 23.VI.1996, J.A.W. Lucas/*Eumerus tarsalis* Loew, det. J.A.W. Lucas {hand written} (PJSA) • MONTENEGRO • 1♀ – Crna Gora, Plužine, Selo Mratinje, 5 jul 2017, 43.268 18.790 870m, 43.265 18.782 960m, leg. Vujić, Aćanski, Kočiš/*Eumerus tarsalis* Loew, 1848, det. A. Vujić 2017 / 16449 (PJSA) • 1♀ – MONTENEGRO, Sv. Nikola, 2 km NNE of Risan, 42°31′41′′N 18°42′20′′E, Elevation 570m, 14-6-2009 11.30-12.30, MP van Zuijen/*Eumerus tauricus* mp2 2011 {hand written}/EUMERUS TARSALIS Loew det. Vujić & Šimić / H0132 (PJSA).

***Eumerus tricolor* (Fabricius, 1798)**

New. SPAIN • 1♂ – ESPAÑA, Valencia, Sª Mariola, Bocairent, Font del Mas dels Arbres, campo de *Thapsia villosa*, 12-VI-2020, Leg.: A. Ricarte / DNA CEUA_S208/CEUA00113561 (CEUA-CIBIO) • 1♀ – ESPAÑA, Valencia, Sª Mariola, Bocairent, Font del Mas dels Arbres, campo de *Thapsia villosa*, 03-VI-2020, Leg.: A. Ricarte/DNA CEUA_S208/CEUA00113562 (CEUA-CIBIO) • 2♂♂ – ESPAÑA, Alicante, Alcoleja, Sierra Aitana, Font del Abre, campo de *Thapsia villosa*, 28-V-2022, Leg.: A. Ricarte/CEUA00113508; 00113509 (CEUA-CIBIO) • 1♀ – ESPAÑA, Alicante, Alcoleja, Sierra Aitana, Font del Abre, campo de *Thapsia villosa*, 28-V-2022, Leg.: A. Ricarte/CEUA00113510 (CEUA-CIBIO) • 1♂ – ESPAÑA, Alicante, Alcoleja, Puerto Tudons, campo de *Thapsia villosa*, 11-VI-2021, Leg.: Z. Nedeljković/CEUA00111069 (CEUA-CIBIO) • 1♀ – ESPAÑA, Alicante, Alcoleja, Puerto Tudons, campo de *Thapsia villosa*, 11-VI-2021, Leg.: Z. Nedeljković/DNA CEUA_S209/CEUA00113560 (CEUA-CIBIO) • 1♂ – ESPAÑA, Alicante, Planes, 38° 47′ 11.68′′N, 0° 17′ 19.25′′O, 567 m, campo de *Thapsia villosa*, 20-V-2021, Leg.: A. Ricarte/CEUA00113563 (CEUA-CIBIO) • 1♀ – Valle de Casares (LEÓN), 14-VI-86, Mª Á. Marcos-García {leg}/*Eumerus tricolor* (Fabricius), Det.: Mª Á. Marcos-García/CEUA00017856 (CEUA-CIBIO) • 1♂ – Vada, SANTANDER, 22-VI-1987, Mª Á. Marcos-García {leg}/*Eumerus tricolor* (Fabricius), Det.: Mª Á. Marcos-García/CEUA00017852 (CEUA-CIBIO) • 2♂♂ – Sotres (SANTANDER), 4-VII-1986, Mª Á. Marcos-García {leg}/*Eumerus tricolor* (Fabricius), Det.: Mª Á. Marcos-García/CEUA00017850; 00017851 (CEUA-CIBIO) • 2♂♂ – Sotres (SANTANDER), 4-VII-1986, Mª Á. Marcos-García {leg}/*Eumerus tricolor* (Fabricius), Det.: Mª Á. Marcos-García/CEUA00017850; 00017851 (CEUA-CIBIO) • 1♂ – SPAIN, Madrid, Perales de Tajuña, UTM: 30T 472 4451, +653m, 15.V.2018, Leg.: P.A. Fidalgo/*Eumerus tricolor* (Fabr.), det. A. v. Eck, 2023 (AET) • 1♂ – Chinchón (MADRID), 18.VI.2021, leg. Piluca AF {hand written}/*Eumerus tricolor* (Fabricius, 1798), Det: P. Aguado-Aranda, 2021/CEUA00113494 (CEUA-CIBIO) • 1♂ – España (Burgos), Covarrubias, N 42°03′24′′ W 3°30′12′′, Eremita San Olav, *29–V–2019*, 916m, leg. Wim Klein/*Eumerus tricolor* (Fabr) ♂ {hand written}, det. A. van Eck, 2020/coll. A. v. Eck (AET).

Revised. SPAIN • 1♂ – Caboalles de Abajo (LEÓN), 13-VI-1986, Mª Á. Marcos-García {leg}/*Eumerus tricolor* (Fabricius), Det.: Mª Á. Marcos-García/CEUA00017846 (CEUA-CIBIO) {published in [45]} • 3♂♂ – Caboalles de Abajo - LEÓN, 2-VI-1987, Mª Á. Marcos-García {leg}/*Eumerus tricolor* (Fabricius), Det.: Mª Á. Marcos-García/CEUA00017847; 00017848; 00017849 (CEUA-CIBIO) {published in [45]} • 1♀ – Morgovejo (LEÓN), 23-VI-87, Mª Á. Marcos-García {leg}/*Eumerus tricolor* (Fabricius), Det.: Mª Á. Marcos-García/CEUA00017854 (CEUA-CIBIO) {published in [45]} • 1♀ – Mirantes de Luna - LEÓN, 3-VI-1987, Mª Á. Marcos-García {leg}/*Eumerus tricolor* (Fabricius), Det.: Mª Á. Marcos-García/CEUA00017855 (CEUA-CIBIO) {published in [45]} • 1♂ – Font Roja (Alcoy) (ALICANTE), 31-V-94, Leg.: L. Sonet/*Eumerus tricolor* Fabricius, 1798, Det: MA. Marcos-García {hand written}/CEUA00017853 (CEUA-CIBIO) {published in [69]} • 1♂ – Font Roja (Alcoy) (ALICANTE), 23-V-94, Leg.: L. Sonet/*Eumerus tricolor* Fabricius, 1798, Det: MA. Marcos-García {hand written}/CEUA00017857 (CEUA-CIBIO) {published in [69]} • 1♂ – Montarco, Arias {leg}/*Eumerus tricolor* (Fabricius, 1798), Det: P. Aguado-Aranda, 2021/MNCN_Ent142892 (MNCN) • 1♀ – Montarco, Dusmet {leg}/*Eumerus tricolor* (Fabricius, 1798), Det. P. Aguado Aranda, 2022/MNCN_Ent301603 (MNCN) {published in [63]} • 1♀ – Aranjuez, G. Mercet {leg}/*Eumerus tricolor* (Fabricius, 1798), Det. P. Aguado Aranda, 2022/MNCN_Ent142893 (MNCN) {published in [63]} • BELGIUM • 1♂ – BELGIË, prov. Namen/Han s. Lesse, 9 – 6 – 1973, J.A.W. Lucas/*Eumerus tricolor* Fabr. det. J.A.W. Lucas (PJSA).
